# Repurposing Si CMOS nonidealities for stochastic and analog image processing

**DOI:** 10.1126/sciadv.aea2328

**Published:** 2026-02-20

**Authors:** Been Kwak, Ryun-Han Koo, Changhyeon Han, Yunho Shin, Joonhyeok Choi, Dongbin Kim, Jongwoo Lee, Jiseong Im, Youngchan Cho, Jong-Ho Lee, Wonjun Shin, Daewoong Kwon

**Affiliations:** ^1^ Department of Electrical Engineering, Hanyang University, Seoul 04763, Republic of Korea.; ^2^ Department of Electrical and Computer Engineering and Inter-university Semiconductor Research Center, Seoul National University, Seoul 08826, Republic of Korea.; ^3^ Department of Semiconductor Convergence Engineering, Sungkyunkwan University, Suwon 16419, Republic of Korea.

## Abstract

Conventional semiconductor device engineering regards intrinsic device nonidealities as reliability concerns to be minimized or eliminated. Here, we demonstrate the strategic repurposing of these nonidealities as functional resources for advanced stochastic analog computing. We leverage two underutilized phenomena—deep-level channel trap-induced generation-recombination (G-R) noise and impact ionization–induced negative differential resistance (NDR) in body current—which have received limited attention compared to the extensively studied 1/*f* noise and monotonic drain current behavior in logic-centric transistors. By exploiting G-R noise with controllable temporal correlation and NDR with an unprecedented peak-to-valley ratio (2.78 × 10^4^) within fully depleted silicon-on-insulator transistors fabricated in industry silicon complementary metal-oxide semiconductor (CMOS) process, we achieve multifunctional analog computation at the single-device level. Our transistor seamlessly performs stochastic encryption, deterministic signal readout, and analog inversion simply through reconfiguration of applied bias conditions, thereby eliminating the need for peripheral random-number generators, dedicated analog inverters, or amplifiers. This approach not only reveals the previously unrecognized computational potential embedded in mature CMOS technologies but also presents a scalable and energy-efficient alternative to architecture based on exotic materials, laying the foundation for next-generation analog computing systems.

## Teaser

A standard CMOS transistor harnesses intrinsic noise and nonlinearity to perform stochastic and analog image processing.

## INTRODUCTION

Historically, continuous scaling of metal-oxide semiconductor field-effect transistors (MOSFETs) has driven advances in computational performance, following Moore’s law (*1*). However, recent technological, physical, and economic limitations indicate the onset of a post-Moore era, requiring exploration beyond traditional MOSFET scaling (*2*). Consequently, notable research has pivoted toward alternative materials and innovative device architectures distinct from conventional silicon complementary metal-oxide semiconductor (CMOS) technology (*3*, *4*). This paradigm shift includes the development of previously unidentified computational frameworks—such as in-memory computing and neuromorphic computing—built upon nonsilicon emerging materials, collectively characterizing the “More-than-Moore” era (*5*, *6*). These frameworks often leverage functional materials such as atomically thin van der Waals materials (*7*).

While these new paradigms predominantly rely on emerging materials—such as transition metal dichalcogenides (*8*), oxychalcogenides (*9*), perovskite oxide (*10*), and Mxenes (*11*)—a parallel line of thought has begun to reexamine the untapped potential of mature Si CMOS technologies as a viable foundation for future computing systems. In this alternative approach, characteristics traditionally regarded as undesirable—so-called nonidealities—are being actively repurposed for previously unknown computational functionalities (table S1). Pazos *et al.* (*12*) recently demonstrated that punch-through impact ionization and hysteretic behavior in standard Si MOSFET—typically regarded as parasitic—can be harnessed via unconventional biasing to emulate both synaptic and neural functions.

Although this approach may appear previously unidentified, these reinterpretations of nonidealities are not unprecedented but rather reflect a recurring theme in semiconductor history. Hot carrier injection and gate oxide tunneling—originally regarded as degradation mechanisms in logic transistors—were later exploited for programming and erasing operations in flash memories (*13*). Gate-induced drain leakage (GIDL), a retention-degrading factor in dynamic random access memory (DRAM), has been repurposed as a hole supply source for erase processes in three-dimensional (3D) VNAND (*14*). These cases exemplify how the deliberate exploitation of nonidealities has repeatedly fueled innovation in semiconductor technology.

**Fig. 1.**
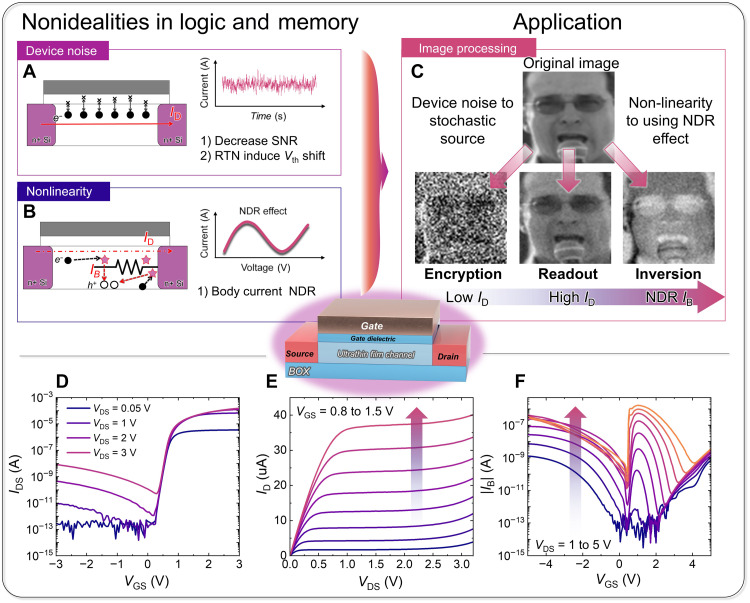
Functional utilization of FD-SOI transistor nonidealities for analog-domain image processing. (**A**) Schematic of device noise, including random telegraph noise (RTN), leads to signal degradation and threshold voltage shifts in conventional logic/memory operations. (**B**) Body current exhibits NDR behavior due to impact ionization under SS region, representing device nonlinearity. (**C**) These nonidealities are repurposed as functional elements for image processing: Stochastic noise is used for encryption at low *I*_D_, high *I*_D_ enables clear readout, and NDR-induced |*I*_B_| enables image inversion [face images from the LFW dataset (*62*)]. (**D**) Transfer characteristics (*I*_D_-*V*_GS_) at various *V*_DS_ values. (**E**) Output characteristics (*I*_D_-*V*_DS_) for increasing *V*_GS_, showing strong current modulation. (**F**) Body current characteristics (|*I*_B_|-*V*_GS_) across *V*_DS_ = 1 to 5 V, highlighting NDR behavior across a wide bias range.

In this article, building on this reinterpretation of nonidealities, we advance stochastic analog computing by leveraging two traditionally undesirable characteristics, low-frequency noise (LFN) and negative differential resistance (NDR) in Si CMOS. We use ultrathin-body fully depleted silicon-on-insulator (FD-SOI) transistors fabricated in a commercial industry, which have been widely adopted in mainstream logic and DRAM applications. Traditionally, LFN in these devices has been considered a reliability concern, as it can degrade sensing accuracy during read operations (Fig. 1A). However, we identify and exploit a distinct form of generation-recombination (G-R) noise originating from deep-level trap dynamics in FD-SOI transistors. Compared to conventional 1/*f* noise caused by gate-oxide defects, this G-R noise offers a superior stochastic source for probabilistic and stochastic computing (*15*, *16*). In parallel, nonlinear current output—typically avoided in conventional logic applications—is harnessed through the body current’s NDR behavior (Fig. 1B). This NDR effect, induced by impact ionization and hole accumulation in the body region, is used to enable analog grayscale inversion. We demonstrate that these nonidealities in a single FD-SOI transistor can perform three key image processing operations—stochastic encryption, deterministic readout, and analog inversion—simply by tuning bias conditions (Fig. 1C). This compact functionality, previously requiring multiple transistors and peripheral circuits, such as random-number generators and analog inverters, can be achieved within a single CMOS-compatible device, offering a scalable and energy-efficient platform for in-sensor image processing (*17*–*19*).

## RESULTS

### Electrical and intrinsic nonideal characteristics in FD-SOI

FD-SOI devices were fabricated in a commercial foundry on a 12-inch wafer using an 80-nm CMOS technology node originally developed for DRAM I/O (input/output) periphery circuits. Detailed fabrication process flow is discussed in the Materials and Methods. Note that the FD-SOI transistors in this work use a thick gate oxide (~5-nm SiO_2_) and a thin channel (~9 nm) with a minimum channel length of 80 nm. They are representative of I/O/peripheral class devices chosen to suppress gate leakage and enable high-voltage pulsed stress. This device is CMOS standard and manufacturable, relying on materials and unit processes compatible with wafer-scale integration.

FD-SOI transistors exhibit conventional electrical characteristics under standard biasing conditions (Fig. 1, D and E). Details of the measurement configuration, 12-inch wafer, FD-SOI device schematic and array patterns are provided in fig. S1. As expected in standard MOSFET operation, the transfer characteristics show a monotonic increase in drain current (*I*_D_) with gate voltage (*V*_GS_), while the output characteristics exhibit clear current saturation behavior with increasing drain voltage (*V*_DS_). In the transfer curve, *I*_D_ increases at large negative *V*_GS_ due to GIDL, and in the output curve, a subtle rise in *I*_D_ at high *V*_DS_ is attributed to weak impact ionization occurring near the drain end of the channel. In stark contrast, the body current (|*I*_B_|) exhibits a strongly nonlinear profile with respect to *V*_GS_ (Fig. 1F) in a range of *V*_DS_ from 1 to 5 V. Most notably, a distinct NDR feature appears, particularly under high *V*_DS_ conditions. Unlike the linear and saturating trends observed in *I*_D_, the nonmonotonic and peaked nature of |*I*_B_| reflects field-enhanced carrier dynamics, including impact ionization in the channel to body region. Both the observed linear and nonlinear characteristics manifest independently of device geometry (fig. S2).

Furthermore, we measured the device-to-device (D2D) variability across 120 FD-SOI transistors distributed on the 12-inch wafer. The individual transfer curves of all devices demonstrate consistent threshold behavior and stable current levels across the wafer (fig. S3). The statistical evaluation of these transfer characteristics shows the distribution of ON-current (*I*_on_) extracted at a fixed gate bias (*V*_GS_ = 1.5 V) and the distribution of subthreshold swing (SS), both exhibiting narrow spreads that confirm the excellent reproducibility of switching characteristics (fig. S4). The |*I*_B_| characteristics of the 120 devices under high drain bias (*V*_DS_ = 4 V) consistently exhibit the distinct NDR profile, demonstrating that this nonlinear |*I*_B_| behavior is robustly observed across many devices (fig. S5).

**Fig. 2.**
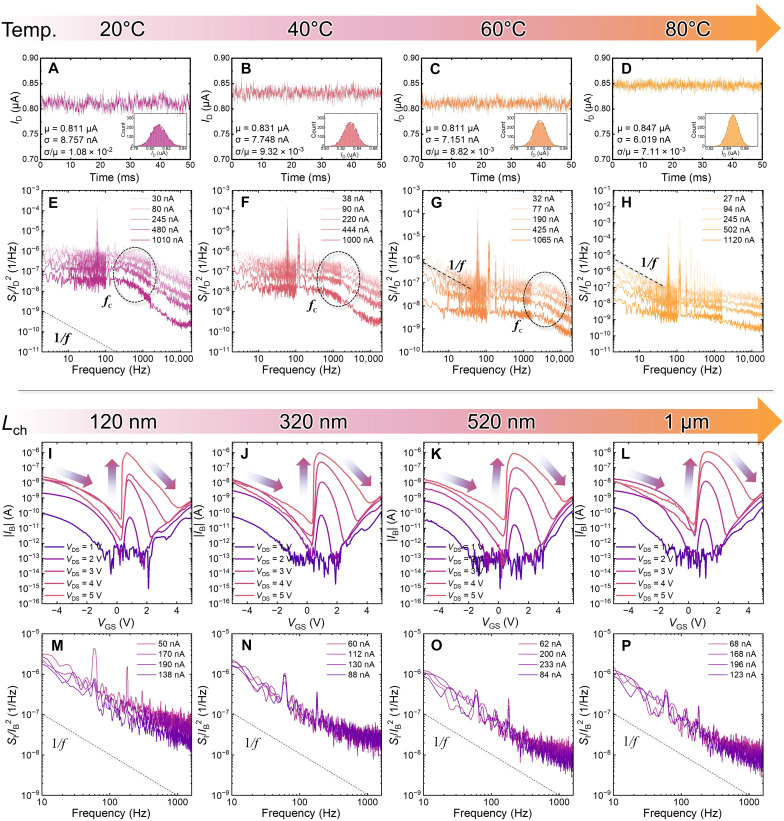
Temperature and device geometry-dependent noise and NDR effects. (**A** to **D**) Time-domain drain current fluctuations measured at various temperatures (20° to 80°C) at 800 nA. Inset of (A) to (D) Corresponding current histograms at 800 nA with average (μ), SD (σ) and μ/σ value at various temperatures (20° to 80 °C). (**E** to **H**) Drain current normalized PSD spectra at various current and temperature. (**I** to **L**) |*I*_B_|-*V*_GS_ measured at *V*_DS_ = 1 to 5 V across devices with different channel lengths (120 nm to 1 μm). (**M** to **P**) Body current normalized PSD spectra various channel lengths (120 nm to 1 μm) with all body current region.

To investigate the intrinsic noise of the FD-SOI devices, we examined current fluctuation behavior in the time domain under various *I*_D_ values (i.e., at 200, 400, and 800 nA) and temperature conditions (Fig. 2, A to D, and fig. S6). Notably, increasing bias (i.e., with increasing *I*_D_) and temperature led to attenuation of current fluctuations in time domain, as reflected by a reduction in amplitude, narrower *I*_D_ histograms, and a decrease in standard deviation. To further elucidate the physical mechanisms of the noise, we transformed the time-domain *I*_D_ fluctuation into the frequency domain by performing power spectral density (PSD) analysis (Fig. 2, E to H). At room temperature, the PSD revealed a distinctive Lorentzian-like plateau with corner frequency (*f*_c_)—features that deviated from classical 1/*f* noise characteristic of conventional bulk MOSFETs, where carrier number fluctuation (CNF) induced by gate dielectric trapping-detrapping events dominates the low-frequency spectrum. Wafer-scale measurements across 120 devices (figs. S7 and S8) demonstrate that the PSD spectra are highly reproducible at 30, 80, 300, and 800 nA, with all devices consistently exhibiting Lorentzian-type noise and only minimal D2D variation, and these characteristics appear independently of device geometry (fig. S9). Here, the *f*_c_ does not change regardless of the *I*_D_ (*V*_GS_), and the noise spectra consistently exhibits Lorentzian features across all bias conditions. Furthermore, as the temperature increased, the *f*_c_ shifted toward higher values and the plateau amplitude diminished, while a 1/*f* spectrum emerged in the low-frequency region (Fig. 2, E to H). These observations suggest the presence of multiple noise sources with distinct activation characteristics, the details of which will be detailed in the next section.

Furthermore, to analyze the origin and consistency of the NDR observed in our FD-SOI devices, we measured the |*I*_B_| as a function of *V*_GS_ across a range of *V*_DS_ from 1 to 5 V. This analysis was conducted on devices with four different channel lengths: 120, 320, 520, and 1 μm (Fig. 2, I to L). The resulting |*I*_B_|-*V*_GS_ characteristics reveal a distinct NDR behavior in all cases. All channel lengths, the |*I*_B_| increases with *V*_GS_, reaches a peak, and then decreases—exhibiting a robust NDR profile that is highly reproducible. Furthermore, even after 100 double-sweep measurements within the *V*_GS_ range of −1 to 3 V at *V*_DS_ = 4 V, the device exhibited minimal variation in its characteristics, demonstrating excellent robustness and stability (fig. S10). The fact that this behavior is preserved regardless of channel length suggests that the underlying mechanism is intrinsic to the device operation and not significantly affected by geometric scaling. Given the strongly nonlinear characteristics observed under NDR biasing, investigating the LFN characteristics offers insight to uncover the microscopic origin governing carrier transport mechanism. LFN measurements were carried out at *V*_DS_ = 4 V for all channel lengths and bias points (Fig. 2, M to P). In every current point, the drain current normalized PSD showed a clear 1/*f* noise. The mechanisms of |*I*_B_| and associated noise characteristics are discussed in detail notes S1 and S2.

### Analysis of LFN characteristics

**Fig. 3.**
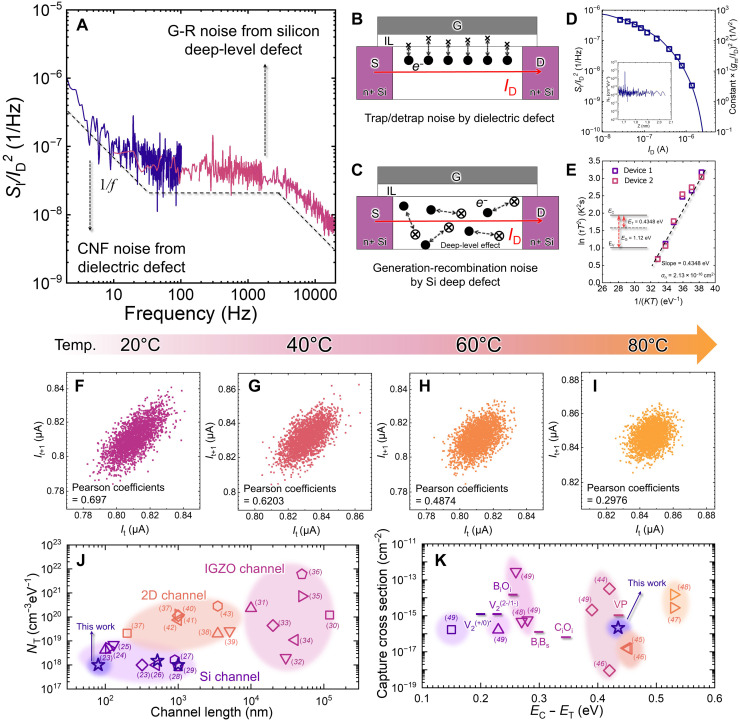
LFN characteristics of FD-SOI. (**A**) Normalized PSD showing an overlap of CNF noise dominated by dielectric traps and G-R noise induced by silicon deep-level defects. (**B**) Schematic illustration of CNF noise mechanism via trapping/detrapping processes in the gate dielectric. (**C**) Schematic illustration of G-R noise mechanism via electron-hole pair generation and recombination through silicon deep-level defects. (**D**) *S*_I_/*I*_D_*^2^* sampled at 10 Hz and (*g*_m_/*I*_D_)^2^ versus *I*_D_. The inset of (D) shows dielectric trap volume density profiling from CNF noise. (**E**) From Arrhenius plot, extracting an activation energy of 0.4348 eV and a capture cross section of 2.13 × 10^−18^ cm^2^, consistent across two measured devices. (**F** to **I**) Temperature-dependent current correlation plots illustrating stochastic fluctuation dynamics. (**J**) Benchmarking of trap volume density (*N*_T_) as a function of channel length, comparing this work with amorphous indium gallium zinc oxide (a-IGZO), 2D material, and previous Si-channel devices. (**K**) Benchmarking of capture cross section versus trap energy level (*E*_C_ − *E*_T_).

The LFN characteristics of the FD-SOI device reveal the presence of two distinct but coexisting noise sources (Fig. 3A). The first is the well-known 1/*f* noise originating from CNF, which is induced by trapping/detrapping processes at defects in gate dielectric (Fig. 3B). The second source arises from G-R processes in deep-level defects within the silicon channel, which give rise to Lorentzian-type spectral components (Fig. 3C). These two mechanisms are superimposed in the observed noise spectrum, with their relative contributions varying with temperature. Note that this study presents the first delineation between CNF noise and G-R noise based on temperature-dependent analysis. The details regarding the theoretical background and modeling approach are provided in note S3 and S4. Note that the potential noise enhancement due to shot noise amplified by the RC network associated with the floating body effects was not considered in this analysis (fig. S11) (*20*).

Temperature-induced suppression of the noise is a hallmark of G-R noise arising from deep-level defects in silicon channel. G-R noise is thermally activated and exhibits Lorentzian spectra whose amplitude decreases with increasing temperature due to the enhanced Shockley-Read-Hall recombination (SRH) rate and reduced occupancy fluctuations of trap states (*21*). These temperature-induced shifts in *f*_c_ exhibit no dependence on channel length, indicating universal behavior across device geometries (figs. S12 and S13). The shift of the *f*_c_ with temperature allows for an Arrhenius plot analysis of the G-R time constant versus 1/*kT*, where the slope reveals the activation energy of the G-R center (*E*_C_ − *E*_T_ = 0.4348 eV), and the intercept determines the capture cross section (σ = 2.13 × 10^−18^ cm^2^) (Fig. 3E, fig. S14, and note S4). Moreover, as temperature increases, there is a change in the form of PSD: from channel G-R noise to 1/*f* noise arising from capture and emission processes at oxide traps.

Based on the CNF-induced 1/*f* noise, the trap volume density (*N*_T_ = 2 × 10^18^ cm^−3^ eV^−1^) can be quantitatively extracted. CNF noise mechanism is generally temperature independent (*22*). Thus, as the temperature increases, the G-R noise diminishes and becomes less prominent, revealing a 1/*f* noise characteristic governed by CNF, with the *N*_T_ remaining unaffected by temperature. The evolution of the current correlation distribution with temperature further supports the transition in dominant noise mechanisms (Fig. 3). At 20 °C, the current correlation plot (Fig. 3F) exhibits a clear positive correlation, indicative of the dominant G-R noise mechanism, which typically shows significant temporal correlation between data points. The Pearson correlation coefficient quantitatively confirms this correlation, with a value of 0.69 at 20°C. As the temperature increases and the G-R noise diminishes, this temporal correlation weakens, with Pearson coefficients decreasing progressively to 0.62 at 40°C, 0.49 at 60°C, and 0.30 at 80°C. At the highest temperature (80°C), the current correlation plot (Fig. 3I) demonstrates significantly reduced temporal correlation, reflecting the dominance of CNF-based 1/*f* noise with minimal correlation between data points. At lower temperatures, the broader spread and elongated shape of the scatter plots indicate significant fluctuation amplitudes and a wider distribution. As the temperature increases, the fluctuations become more isotropic, suggesting a transition toward CNF-dominated 1/*f* noise originating from dielectric defects. This temperature-dependent behavior in correlation profile reinforces the presence of distinct, thermally activated noise sources contributing to the overall LFN behavior.

To contextualize the trap characteristics of our FD-SOI devices, we benchmarked the extracted *N*_T_ as a function of channel length across various semiconductor platforms (Fig. 3J). State-of-the-art CMOS transistors fabricated on silicon exhibit the shortest channel lengths and among the lowest recorded trap densities (*23*–*29*), reflecting decades of process optimization and mature gate dielectric integration (fig. S15). Against this landscape, our FD-SOI devices, fabricated at an 80-nm technology node—a commercially mature platform widely adopted in the semiconductor industry (fig. S16)—achieve a notably lower trap volume density compared to indium gallium zinc oxide (IGZO) (*30*–*36*) and 2D semiconductors (tables S2 and S3) (*37*–*43*). This result highlights the exceptional interface and dielectric quality attainable through fully depleted SOI architectures. Note that although the FETs exhibit a low *N*_T_, this does not hinder the manifestation of the stochastic behavior exploited in this work. This is because the stochastic analog computing functionality relies on G-R noise arising from deep-level channel traps, rather than oxide traps in the gate dielectric. Furthermore, to assess the physical nature and impact of the dominant traps in our FD-SOI devices, we extracted their activation energy (*E*_C_ − *E*_T_) and capture cross section (σ) from the temperature dependence of the G-R noise spectra. Figure 3K shows a benchmarking map of these trap parameters, where the *x* axis denotes the trap energy level (*E*_C_ − *E*_T_) and the *y* axis indicates the capture cross section (*44*–*49*). A benchmarking table comparing activation energy and capture cross section between previous CMOS studies and this work is provided in table S4.

### Investigation of NDR characteristics

**Fig. 4.**
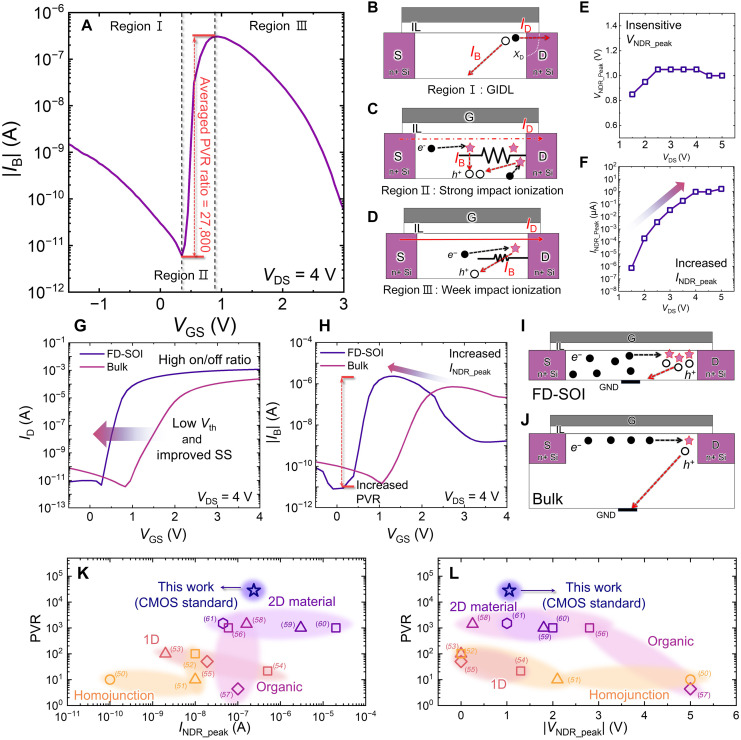
NDR behavior. (**A**) |*I*_B_| versus *V*_GS_ curve at *V*_DS_ = 4 V. An average peak-to-valley current ratio (PVR) of 27,800 across 120 devices is achieved. (**B** to **D**) Schematics illustrating the mechanisms in each region: (B) band-to-band tunneling-induced GIDL current, (C) strong impact ionization under high lateral electric field conditions, and (D) suppressed impact ionization after channel inversion. (**E**) Insensitive *V*_NDR_peak_ with various *V*_DS_ (**F**) *V*_DS_-dependent increase in *I*_NDR_peak_. (**G**) Simulated transfer and (**H**) |*I*_B_| characteristics of FD-SOI and bulk MOSFET. Schematic illustration of the impact ionization mechanism in (**I**) FD-SOI and (**J**) bulk MOSFET. Benchmarking of PVR versus (**K**) *I*_NDR_peak_ and (**L**) ∣*V*_NDR_peak_∣ across various NDR technologies, including homojunction, 1D materials, organic semiconductors, and 2D materials.

The observed |*I*_B_| characteristics can be categorized into three distinct regimes based on *V*_GS_ and the underlying carrier transport mechanisms (Fig. 4A). In region I, |*I*_B_| is dominated by GIDL, driven by band-to-band tunneling in the drain-to-body overlap region under low gate bias (Fig. 4B). In region II, corresponding to the subthreshold regime, the |*I*_B_| rises sharply as the lateral electric field enables impact ionization of the channel. This marks the onset of NDR behavior as electron-hole pair generation becomes significant (Fig. 4C). In region III, strong inversion reduces channel resistance, thereby suppressing impact ionization. As a result, the |*I*_B_| decreases with increasing gate voltage, giving rise to the observed NDR profile (Fig. 4D). A detailed explanation of the physical mechanisms and temperature dependency governing each region is provided in note S1 and fig. S17. These results confirm that the observed NDR is a stable and intrinsic feature of the device, likely arising from field-dependent carrier transport phenomena, such as impact ionization occurring near the channel-body junction. Notably, the LFN measured across all current levels under NDR conditions exhibits a consistent 1/*f* spectral shape with comparable amplitude. The underlying physical mechanisms of these characteristics are discussed in detail in note S2.

NDR is usually characterized by peak-to-valley ratio (PVR), defined as peak current (*I*_NDR_peak_)/valley current (*I*_NDR_valley_). A higher PVR indicates a more pronounced NDR effect. In FD-SOI devices, the average NDR characteristics extracted from 120 devices yielded a remarkably high PVR of 2.78 × 10^4^ at *V*_DS_ = 4 V, underscoring the sharpness and reliability of the NDR transition (fig. S18). The gate voltage at which the NDR peak occurs (*V*_NDR_peak_) remains nearly constant across different drain bias levels (Fig. 4E). This bias insensitivity arises because the NDR phenomenon is confined to the subthreshold region, where the channel is not yet in strong inversion, and thus, the electrostatic gate control dominates over the drain-induced field modulation. In contrast, the *I*_NDR_peak_ increases steadily with increasing *V*_D__S_, reflecting the enhanced probability of impact ionization under higher lateral electric fields in the channel (Fig. 4F). As *V*_D__S_ increases, electrons gain more kinetic energy, leading to more frequent carrier multiplication events and a corresponding rise in the magnitude of *I*_NDR_peak_. Accordingly, the NDR characteristics of the |*I*_B_| exhibit temperature-dependent variations (figs. S17 and S19).

Through TCAD (technology computer-aided design) simulations, we conducted a comparative analysis of the body current NDR behavior in FD-SOI and conventional bulk MOSFET devices. The detailed simulation methodology and calibration are described in the Materials and Methods, fig. S20, and table S5. As revealed by the transfer characteristics, the FD-SOI transistor exhibits superior gate controllability compared to its bulk counterpart, resulting in a higher on/off current ratio, lower SS, and reduced threshold voltage (*V*_th_) (Fig. 4G). Notably, the NDR region of the body current corresponds to the SS (subthreshold swing) regime of the MOSFET. Because of the inherently lower *V*_th_ of the FD-SOI structure, NDR characteristics can be effectively realized at lower gate voltages (Fig. 4H). Furthermore, the steeper SS enables a greater number of carriers to contribute to impact ionization at the same *V*_th_ condition (e.g., via work-function tuning), thereby enhancing the NDR current (Fig. 4, I and J). This leads to an improved PVR, positioning FD-SOI transistors as promising candidates for high-performance NDR-based electronic devices. Moreover, based on TCAD simulations, fig. S21 shows that changing the device width and length under an identical stack yields essentially the same NDR behavior. This supports the view that the effect is governed by the underlying device physics rather than a particular geometry choice.

To compare the performance and integration potential of our FD-SOI–based NDR device, we benchmarked it against previously reported NDR devices fabricated using homojunction semiconductors (*50*–*52*), 1D nanostructures (*53*–*55*), organic materials (*56*–*58*), and 2D channels (*59*–*61*)—all operating within a ±5-V bias window. We constructed two performance maps: one plotting NDR peak current against PVR and another plotting NDR peak voltage versus PVR (Fig. 4, K and L). The use of exotic material systems—such as organic films, 1D, organic and layered 2D materials—renders these devices incompatible with standard CMOS manufacturing processes, making large-scale integration and practical deployment challenging. In contrast, our FD-SOI device uses body current modulation to realize NDR behavior using a fully CMOS-compatible platform. Among all benchmarked devices, it exhibits the highest PVR, even when compared to those based on advanced material systems. Furthermore, the NDR peak voltage in our device is significantly lower, enabling operation under reduced power conditions. This combination of record-high PVR and low operating voltage, realized within a standard silicon process, sets our device apart as a scalable and integration-ready solution for NDR application system.

### Image processing

**Fig. 5.**
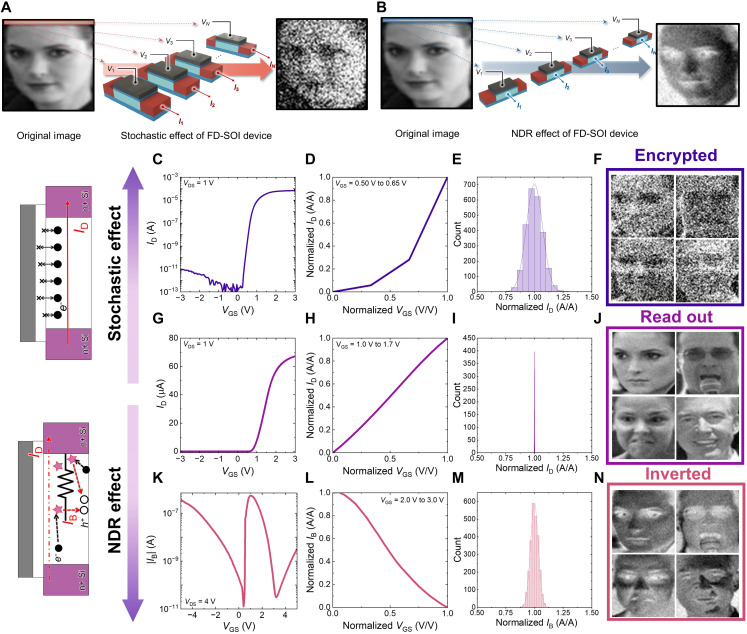
Multifunctional image processing enabled by intrinsic stochasticity and NDR behavior. (**A** and **B**) Compared two distinct effects of FD-SOI devices when processing visual input: (A) stochastic behavior versus (B) NDR response. (**C** to **F**) Stochastic encryption using inherent transistor noise. (C) Transfer characteristics measured at *V*_DS_ = 1 V (log scale). (D) Corresponding normalized transfer characteristics defining the encryption window. (E) Current distribution histogram showing a broad Gaussian profile suitable for encryption. (F) Representative images demonstrating effective noise-driven encryption, rendering original images indistinguishable. (**G** to **J**) Deterministic readout with suppressed noise. (G) Transfer characteristics measured at *V*_DS_ = 1 V (linear scale). (H) Normalized transfer characteristics highlighting ideal linearity. (I) Sharply peaked current distribution histogram confirming stable readout. (J) Reconstructed facial images demonstrating faithful readout capability. (**K** to **N**) Analog inversion enabled by intrinsic NDR characteristics. (K) |*I*_B_| versus *V*_GS_ characteristics at *V*_DS_ = 4 V, displaying pronounced NDR with a high PVR (~2.78 × 10^4^). (L) Normalized NDR transfer characteristics with clear negative slope enabling intrinsic analog inversion. (M) Narrow current distribution histogram indicating stable operation under NDR conditions. (N) Grayscale-inverted images produced directly by intrinsic transistor response, confirming successful analog inversion without additional circuitry. Note that face images are sourced from the LFW dataset (*62*).

Conventional hardware implementations of image processing tasks such as encryption, pixel inversion, and accurate data readout generally require multiple transistors and complex peripheral circuits, including random-number generators, analog inverters, and amplifiers (*17*–*19*). Departing from this conventional approach, here we demonstrate that a standard, industry-fabricated FD-SOI transistor can inherently achieve all three functionalities—image encryption, inversion, and faithful readout—within a single device, notably simplifying the pixel-level architecture and markedly reducing both complexity and energy consumption. This compact integration is enabled by exploiting two intrinsic device characteristics: the stochastic noise of the drain current arising from G-R events (Fig. 5A), and the pronounced NDR behavior in the transistor’s body current (Fig. 5B), resulting from intensity of impact ionization within the body region. To clarify, the 28 × 28 and 64 × 64 image demonstrations were not obtained by physically wiring hundreds or thousands of transistors, but rather by simulations based on pseudo-arrays generated from measured device statistics using a measured-device-in-the-loop approach. The detailed rationale and correlation-aware Monte Carlo procedure underlying these pseudo-arrays are provided in note S5 and figs. S22 to S24.

The concept of single transistor multifunctionality is illustrated using a representative facial image from the LFW (Labeled Faces in the Wild) dataset, along with simplified cross-sectional schematics depicting the two key operating mechanisms (Fig. 5, A and B). Each pixel of the input image is mapped onto the gate terminal of an individual FD-SOI transistor, enabling direct encoding of image data by measuring either drain or body currents. When a low gate voltage is applied, stochastic G-R noise significantly affects the drain current, resulting in random fluctuations that effectively encrypt the original image. Because G-R noise has a Lorentzian spectrum with a finite correlation time, its temporal correlations are larger than those of conventional 1/*f* noise, providing a tunable stochastic source for encryption and other probabilistic operations (*15*, *16*). Increasing the gate voltage suppresses these stochastic fluctuations, allowing accurate reconstruction and decryption of the original image. Conversely, encoding via the body current exploits the intrinsic NDR response, directly enabling inversion of the image pixel values. Thus, multifunctional image processing—including encryption, accurate readout, and inversion—can be achieved by simply selecting appropriate bias conditions and measuring the corresponding transistor currents (Materials and Methods).

The transfer characteristics at a fixed drain voltage (*V*_DS_ = 1 V) in log scale is shown in Fig. 5C. At gate biases below ~0.65 V, discrete G-R noise events cause distinct fluctuations as evident from the serrations in the transfer characteristic. This strongly stochastic behavior defines the encryption window. Specifically, the bias region from *V*_GS_ = 0.5 V to 0.65 V is used, where the current response is inherently noisy enough to enable encryption simply by reading instantaneous current values. Normalizing the *V*_GS_ and *I*_D_ within this encryption window to a scale of 0 to 1, the corresponding normalized transfer characteristics are plotted in Fig. 5D. The resulting histogram shown in Fig. 5E quantitatively illustrates the stochastic drain current fluctuations captured from transient measurements (sampled at 3.2 kHz over 5 s). The broad Gaussian-shaped histogram (relative SD of ~7.1%) originates from random charge-carrier trapping and detrapping at deep-level defects in the channel. Encoding pixel grayscale intensities from four facial images from the LFW dataset into this stochastic regime results in effectively scrambled output images, as displayed in Fig. 5F. The original images become indistinguishable, demonstrating effective noise-driven encryption without external random-number generation circuitry.

Increasing the gate voltage to ~1.0 to 1.7 V moves the device into a deterministic operation mode, the readout window, where transfer characteristics exhibit a smooth, monotonic, and noise-free response over several orders of magnitude (Fig. 5G). The corresponding normalized transfer curve shows a nearly ideal linear relationship indicative of highly deterministic transconductance suitable for accurate image sensing (Fig. 5H). The sharply peaked histogram confirms significant suppression of stochastic fluctuations (relative standard deviation < 0.15%), indicating stable current output (Fig. 5I). Consequently, the device faithfully converts input pixel voltages into proportional output currents, enabling accurate reconstruction of the original LFW images as shown in Fig. 5J, without requiring additional amplification or complex calibration circuitry.

Encoding via the transistor’s |*I*_B_|, enabled by higher biases (*V*_DS_ = 4 V), provides intrinsic grayscale inversion due to its pronounced NDR characteristic. The |*I*_B_| versus *V*_GS_ characteristics exhibit a rapid initial increase, peaking sharply near *V*_GS_ ≈ 0.8 V and subsequently decreasing by more than four orders of magnitude, achieving a remarkably high PVR (~2.78 × 10^4^) (Fig. 5K). This intrinsic NDR behavior arises from impact ionization near the drain region, creating an inverse relationship between input voltage and output current. The normalized NDR transfer curve emphasizes its pronounced negative slope suitable for intrinsic grayscale inversion (Fig. 5L). Despite the avalanche nature of impact ionization, fast recombination dynamics average out LFN, yielding a narrow current distribution as illustrated in the corresponding histogram (Fig. 5M) (relative standard deviation ~3%). When pixel voltages of LFW facial images are applied to this NDR inversion regime, the transistor directly produces grayscale-inverted output images, as clearly demonstrated in Fig. 5N, without external analog inversion circuitry or additional transistor stages.

By integrating these three intrinsic operating modes—noise-driven stochastic encryption (Fig. 5, C to F), deterministic readout (Fig. 5, G to J), and analog inversion via body current NDR (Fig. 5, K to N)—within a single FD-SOI transistor, this approach substantially simplifies the pixel-level circuit design, significantly reduces power consumption, and eliminates area overhead. Unlike conventional multidevice architectures or platforms relying on specialized semiconductor materials, this single-transistor solution offers a compact, energy-efficient, and fully CMOS-compatible hardware platform for multifunctional in-sensor image processing.

## DISCUSSION

We have demonstrated a previously unidentified approach to harnessing traditionally undesirable characteristics of mature CMOS technologies—LFN and nonlinear output current response (NDR) in FD-SOI transistors—for advanced analog and stochastic computing. By reinterpreting these nonidealities as functional resources rather than reliability concerns, we successfully implemented multifunctional image processing operations—including stochastic encryption, stable readout, and analog inversion—within a single-transistor framework. This work highlights the largely untapped potential of mature silicon platforms and presents a compelling alternative to the use of emerging materials, which often face critical challenges in large-scale integration and process uniformity. Ultimately, our findings advocate for a paradigm shift: by embracing and repurposing inherent device behaviors, next-generation computing technologies can be realized using existing, commercially proven semiconductor processes—bridging the gap between today’s mature technologies and tomorrow’s computational demands.

## MATERIALS AND METHODS

### Fabrication process

FD-SOI transistors were fabricated on 300-mm SOI wafers with a 9-nm silicon channel layer and a 200-nm buried oxide (BOX). The fabrication process began with wafer preparation and prephoto cleaning, followed by photolithography and dry etching steps to define the active regions. A thin gate oxide layer (~5-nm SiO_2_) was then deposited by atomic layer deposition (ALD), and a 50-nm phosphorus-doped poly-Si layer was deposited by low-pressure chemical vapor deposition to form the gate electrode. After gate patterning and etching, source/drain regions were formed by phosphorus and boron ion implantations for n^+^ doping. Dopant activation was performed by rapid thermal annealing at 1050°C for 30 s under N_2_ ambient. Subsequently, an interlayer dielectric oxide was deposited and annealed, followed by chemical mechanical polishing (CMP) for planarization. Contact holes were defined by photolithography and dry etching, and doped poly-Si plugs were deposited and planarized by CMP and etch-back processes. Tungsten (W) was then deposited for contact metallization, followed by CMP and etch-back to ensure planar surface morphology. Last, the first metal (Metal-1) layer was deposited, patterned, and etched to complete the device structure. All fabrication steps were carried out using an 80-nm CMOS-compatible process flow.

### LFN measurement

To investigate the LFN characteristics of the FD-SOI device, we used a precise measurement setup incorporating a semiconductor parameter analyzer (B1500A), a low-noise current amplifier (SR570), and a signal analyzer (35670A). The measurement methodology is designed to capture the drain current fluctuations with high sensitivity and accurately extract the PSD through fast Fourier transform (FFT) analysis. In our setup, the gate bias is applied using the B1500A, ensuring precise control over the transistor’s operating point. Simultaneously, the drain bias is applied through SR570, which not only supplies the required voltage but also functions as a low-noise current amplifier to enhance the signal-to-noise ratio. The amplified drain current signal is then fed into the 35670A signal analyzer, which processes the time-domain fluctuations and extracts the frequency-dependent noise characteristics using FFT-based PSD calculations. The selection of this measurement technique is based on the fundamental principle that, in FETs, the primary source of signal fluctuations originates from variations in the drain current. Consequently, monitoring the drain current noise spectrum provides valuable insight into the underlying noise mechanisms, including carrier G-R noise and trapping/detrapping processes noise contributions.

In this study, the frequency range for LFN measurements was carefully chosen to cover the wide spectrum. Note that these results are obtained by performing three separate measurements of the PSD within different frequency ranges: 2 Hz < *f* < 202 Hz, 10 Hz < *f* < 1610 Hz, and 1000 Hz < *f* < 20,000 Hz, all using the same gain for the low noise current preamplifier. Furthermore, since G-R noise is known to be highly sensitive to temperature variations, we conducted noise measurements at multiple temperatures ranging from 20° to 100°C. This allowed us to systematically separate and analyze the G-R and CNF noise components across different temperature regimes.

### Electrical characteristic measurement

To evaluate the fundamental switching characteristics of the device, dc current-voltage (*I*-*V*) measurements were performed using the Agilent B1500A Semiconductor Parameter Analyzer equipped with precision Source Measure Unit modules. For transient analysis in the time domain, the Waveform Generator/Fast Measurement Unit (B1530A) was used, enabling high-resolution capture of dynamic switching behavior under fast voltage pulsing conditions. In addition, array-level electrical characterization was carried out using an E5250A switching matrix connected to a probe card.

### Image processing simulation procedure

Face photographs (LFW) sourced from the LFW dataset (*62*) were converted to 28 × 28 or 64 × 64 grayscale arrays. Pixel intensities (*p*) were normalized to lie between 0 (black) and 1 (white). Each pixel value was then translated into a gate-bias voltage (*V*_Pixel_) applied individually to FD-SOI transistors, and the resulting drain current (*I*_D,Pixel_)—or body current (*I*_B,Pixel_) in the inversion mode—was read and remapped to generate processed pixel intensities as follows.

For image encryption (high-noise window, *V*_GS_ = 0.50 to 0.65 V), pixel intensities were mapped as *V*_Pixel_ = 0.50 V + 0.15 V × *p*. Generation-recombination noise within this bias range caused stochastic variations in the drain current. Encrypted pixel intensities were obtained by normalizing the measured currents according to *p*_Enc_ = [*I*_D,Pixel_ − *I*_D_(0.50 V)]/[*I*_D_(0.65 V) − *I*_D_(0.50 V)], resulting in each read producing an encrypted image.

For image read-out (low-noise window, *V*_GS_ = 1.00–1.70 V), pixel intensities were mapped using *V*_Pixel_ = 1.00 V + 0.70 V × *p*. The resulting drain currents, having minimal noise in this range, were directly remapped back to grayscale as *p*_Read_ = [*I*_D,Pixel_ − *I*_D_(1.00 V)]/[*I*_D_(1.70 V) − *I*_D_(1.00 V)], providing accurate image reconstruction with residual noise below 0.3%.

Image inversion used the NDR regime of the transistor’s body current (*V*_GS_ = 2.00–3.00 V, *V*_DS_ = 4 V). Pixel intensities were translated to gate voltages as *V*_Pixel_ = 2.00 V + 1.00 V × *p*. Then inverted grayscale pixel intensities were computed by *p*_Inv_ = [*I*_B,Pixel_ − *I*_B_(2.00 V)]/[*I*_B_(3.00 V) − *I*_B_(2.00 V)], effectively flipping dark pixels to bright and vice versa.

### Device TCAD simulation

Device simulations were carried out using Synopsys Sentaurus TCAD. An FD-SOI structure with a gate length of 120 nm and a width of 10 μm was modeled. The source and drain regions were doped with arsenic at a concentration of 1 × 10^20^ cm^−3^, and a raised source/drain structure was applied. The channel region was intrinsic. Simulations were performed by solving the Poisson equation coupled with the drift-diffusion transport model. Quantum confinement effects were included using the density gradient model. Impact ionization was modeled using the van Overstraeten de Man model.

## Supplementary Materials

### This PDF file includes:

Figs. S1 to S24 Tables S1 to S5 Supplementary Notes S1 to S5 References
