# Supplementary material for: Repurposing Si CMOS nonidealities for stochastic and analog image processing
Source: Sci Adv. 2026 Feb 20;12(8):eaea2328. doi: 10.1126/sciadv.aea2328 (PMC12922738; doi:10.1126/sciadv.aea2328)
Supplement: Supplementary file 1 — Figs. S1 to S24 Tables S1 to S5 Supplementary Notes S1 to S5 References [file sciadv.aea2328_sm.pdf]

Science Advances
AAAS

Supplementary Materials for

Repurposing Si CMOS nonidealities for stochastic and analog image processing

Been Kwak *et al.*

Corresponding author: Wonjun Shin, swj0107@skku.edu; Daewoong Kwon, dw79kwon@hanyang.ac.kr

Sci. Adv. **12**, eaea2328 (2026)
DOI: 10.1126/sciadv.aea2328

This PDF file includes:

Figs. S1 to S24
Tables S1 to S5
Supplementary Notes S1 to S5
References

Supplementary Figures

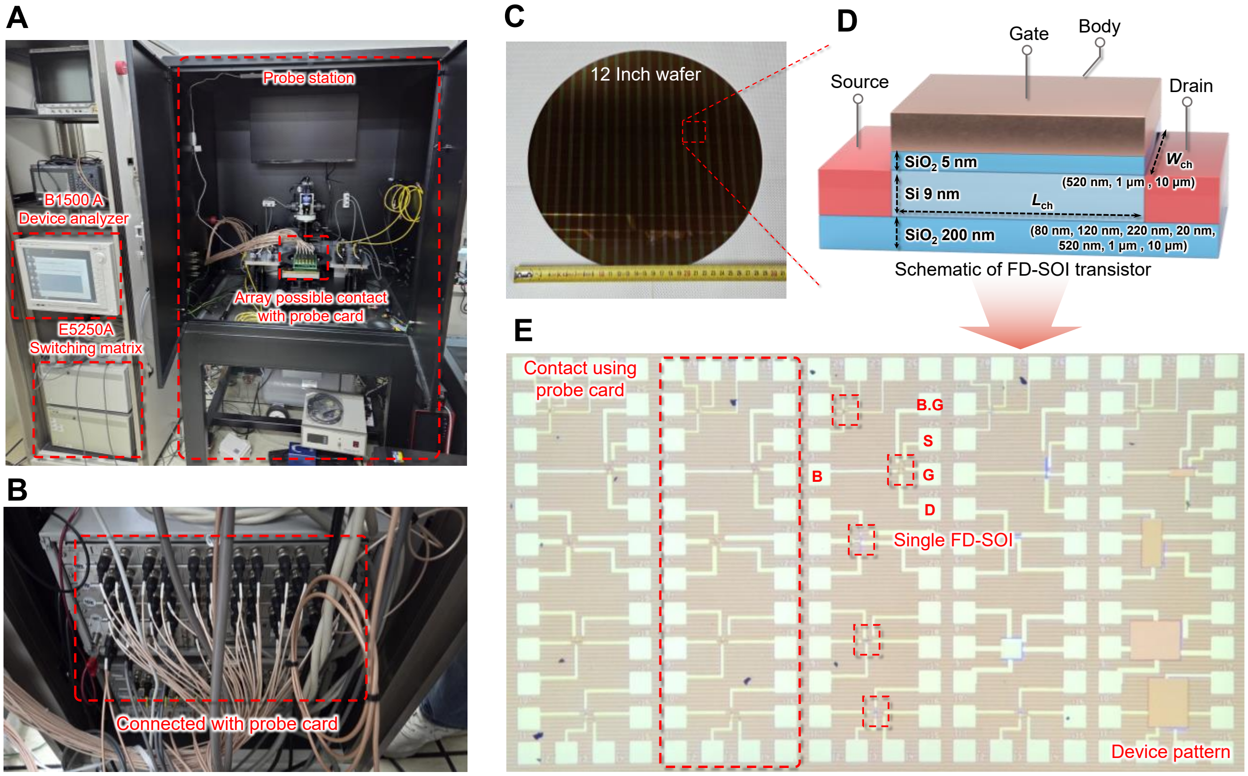

Fig. S1. Measurement and device information. (**A**) Measurement environment, (**B**) switching matrix, (**C**) wafer, (**D**) schematic of FD-SOI and (**E**) single device configuration of fabricated FD-SOI devices.

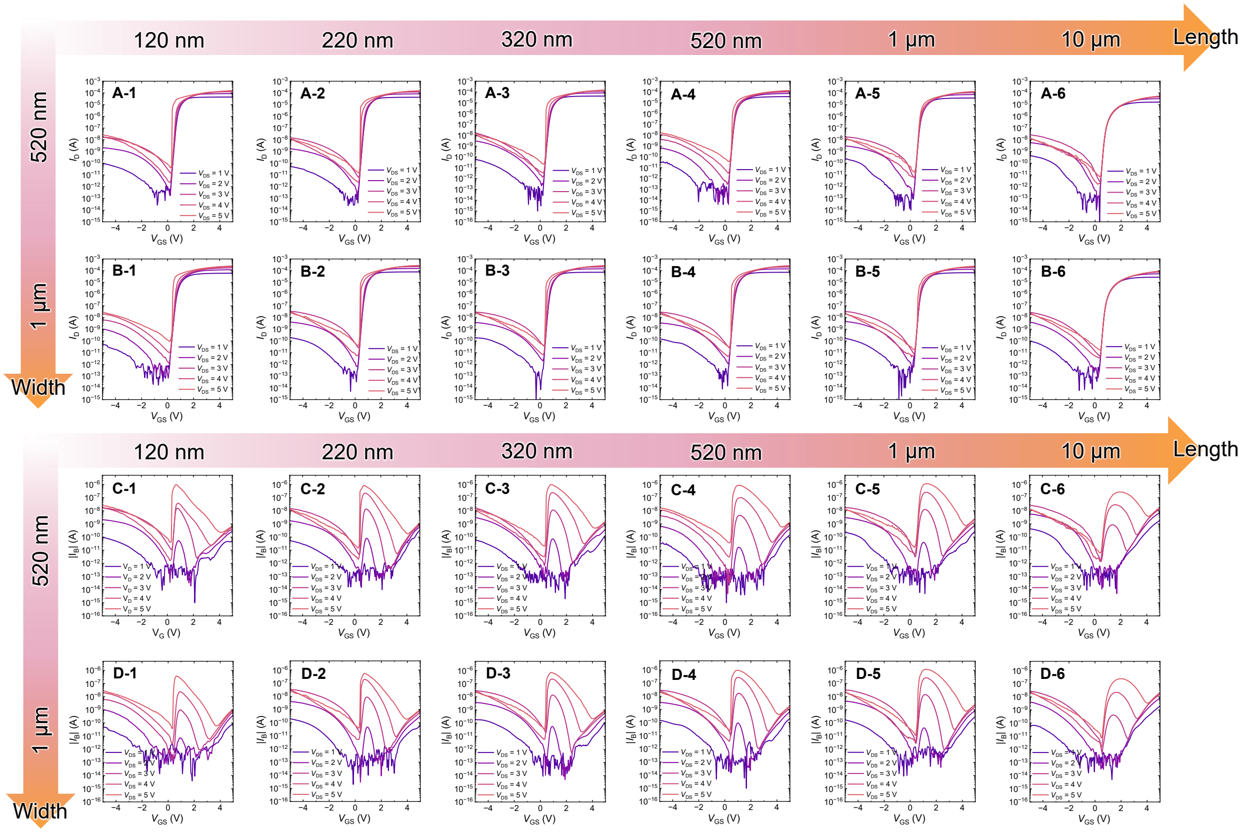

Fig. S2. Transfer characteristics and body current behavior across different device areas. I_D-V_{GS} for devices with channel lengths ranging from 120 nm to 10 μm and widths of (**A-1** to **A-6**) 520 nm and (**B-1** to **B-6**) 1 μm. $|I_B|$-V_{GS} for devices with channel lengths ranging from 120 nm to 10 μm and widths of (**C-1** to **C-6**) 520 nm and (**D-1** to **D-6**) 1 μm.

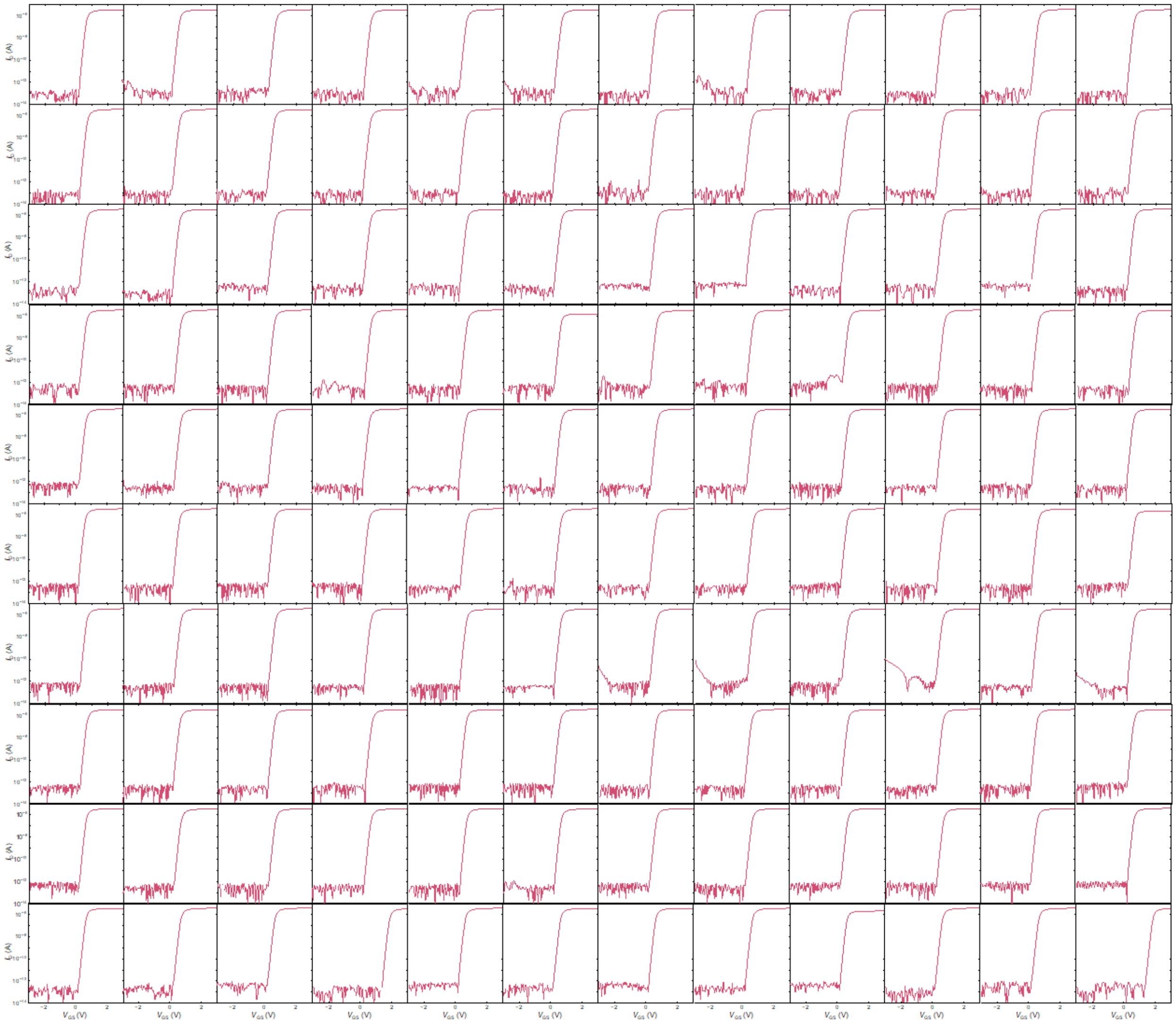

Fig. S3. Transfer characteristics of 120 FD-SOI transistors measured across a 12-inch wafer at V_{DS} of 0.05 V.

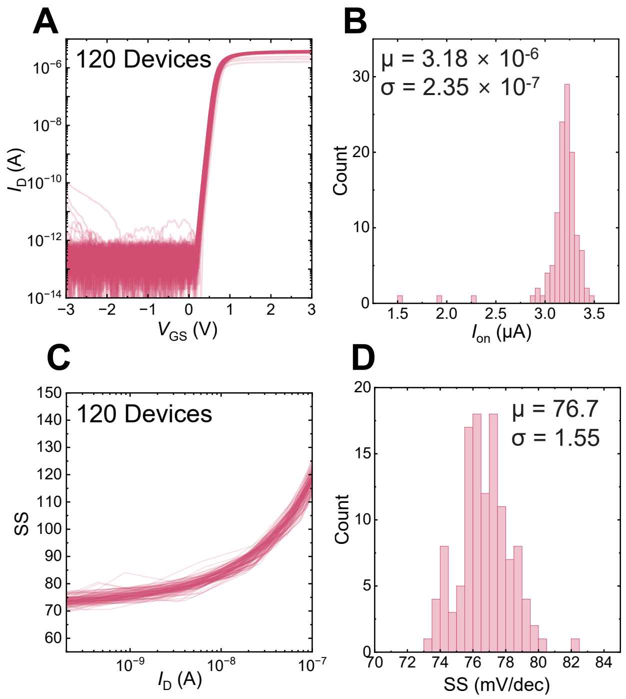

Fig. S4. Statistical evaluation of electrical characteristics across 120 FD-SOI devices. (**A**) Transfer characteristics of 120 FD-SOI devices measured at V_{DS} = 0.05 V. (**B**) Statistical distribution of the I_{on} extracted at V_{GS} = 1.5 V, with a mean value of 3.18 × 10^{-6} A and a standard deviation of 2.35 × 10^{-7} A. (**C**) Subthreshold swing (SS) characteristics as a function of I_D for 120 FD-SOI devices, (**D**) Histogram of SS, showing a narrow distribution with a mean of 76.7 mV/dec and a standard deviation of 1.55 mV/dec

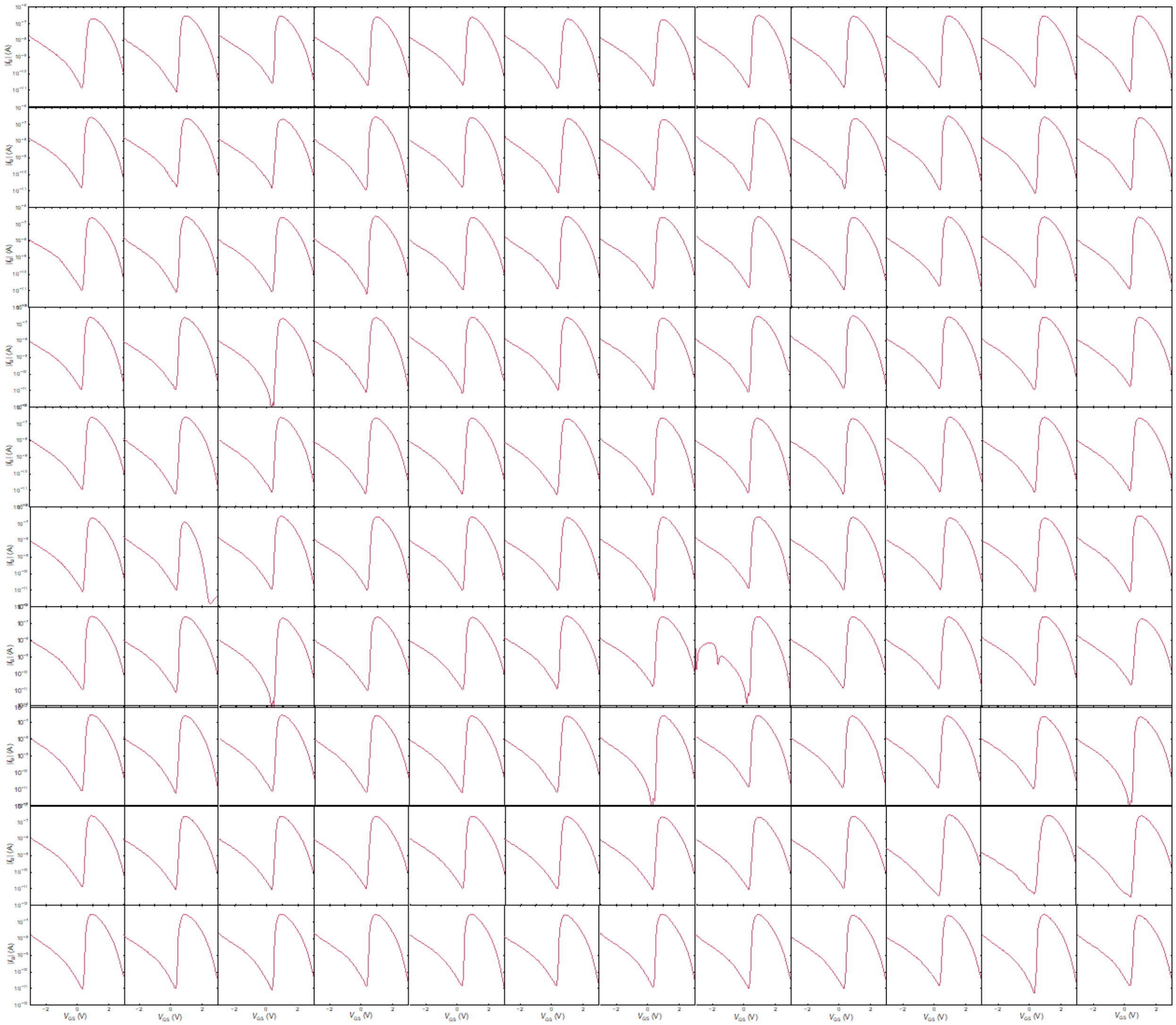

Fig. S5. NDR characteristics of 120 FD-SOI transistors measured across a 12-inch wafer at V_{DS} of 4 V.

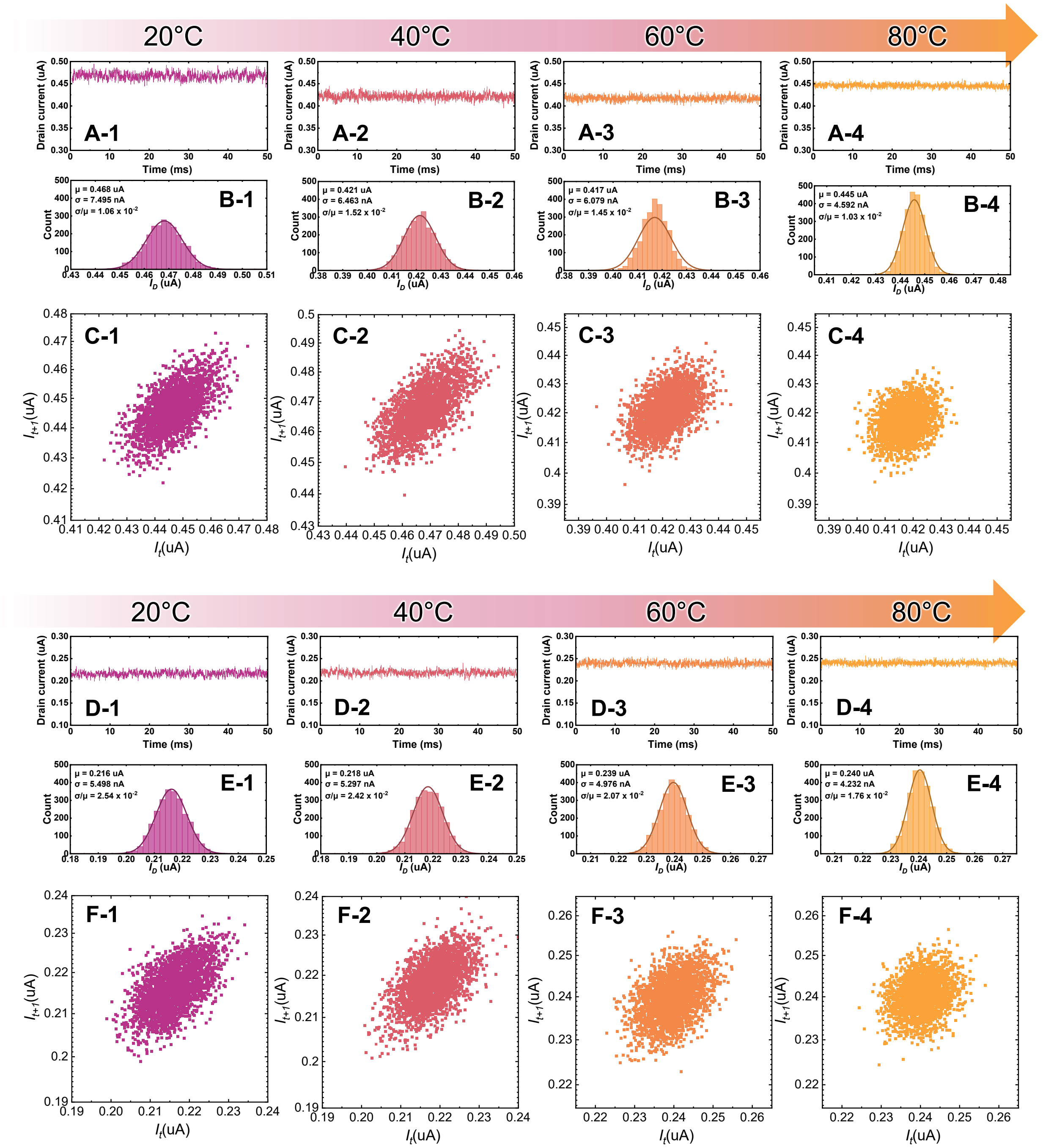

Fig. S6. Time-domain current fluctuations, histograms, and correlation analysis at 200 nA and 400 nA under varying temperatures. Time-domain drain current fluctuations were measured at fixed drain current levels of (**A-1** to **A-4**) 200 nA and (**D-1** to **D-4**) 400 nA under varying temperature conditions. As the temperature increased, the amplitude of the current fluctuations in the time domain decreased noticeably. Correspondingly, the standard deviation of the current distributions, obtained from histogram analysis, also exhibited a clear downward trend with rising temperature at (**B-1** to **B-4**) 200 nA and (**E-1** to **E-4**) 400 nA. In addition, correlation analysis revealed that the spread of current correlations diminished with increasing temperature, indicating a reduction in the temporal variability and noise magnitude at (**C-1** to **C-4**) 200 nA and (**F-1** to **F-4**) 400 nA. These observations consistently support the transition from G–R noise dominated behavior at low temperatures to CNF behavior at elevated temperatures.

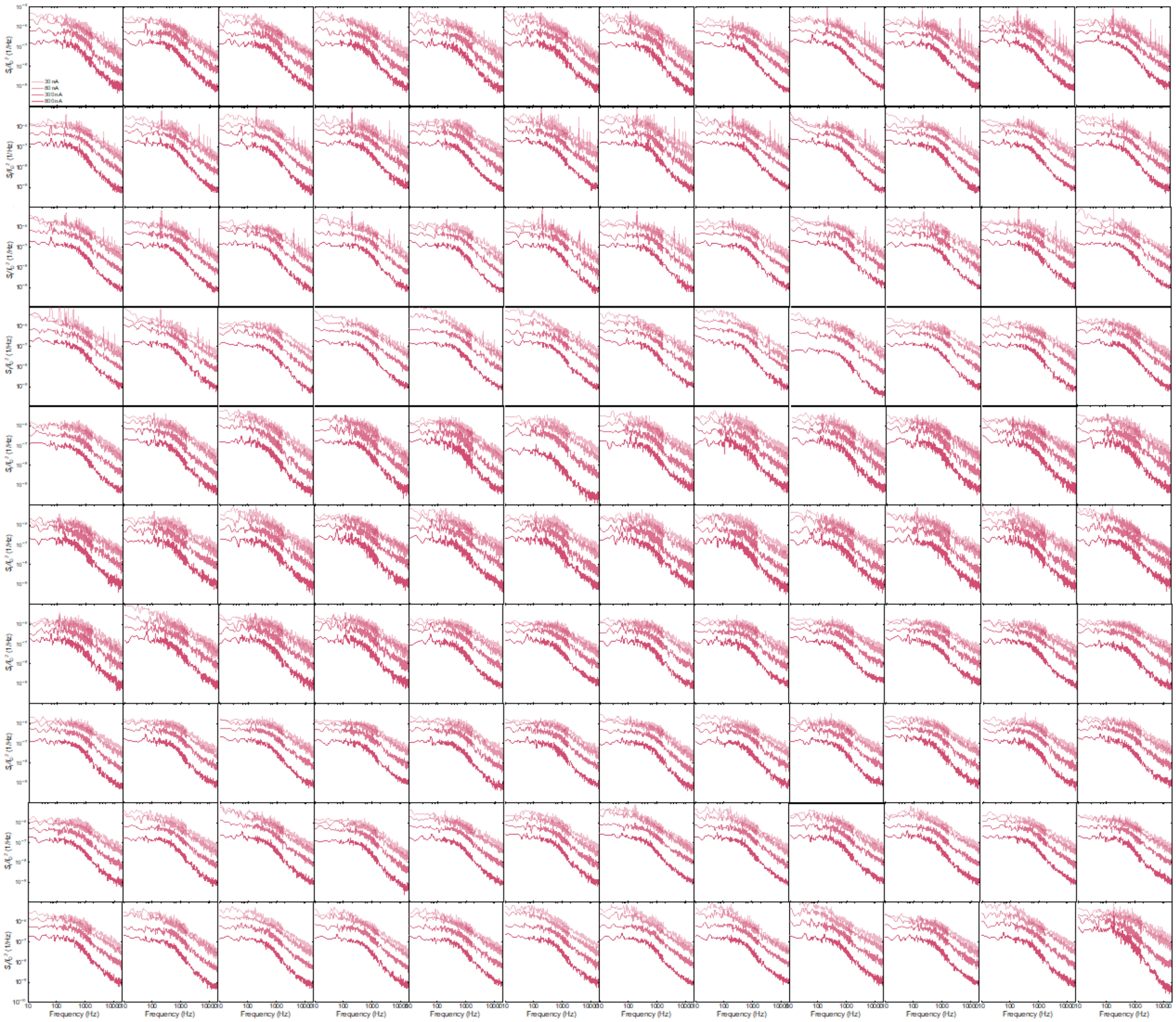

Fig. S7. PSD characteristics of 120 FD-SOI transistors across a 12-inch wafer evaluated at I_D levels of 30, 80, 300, and 800 nA.

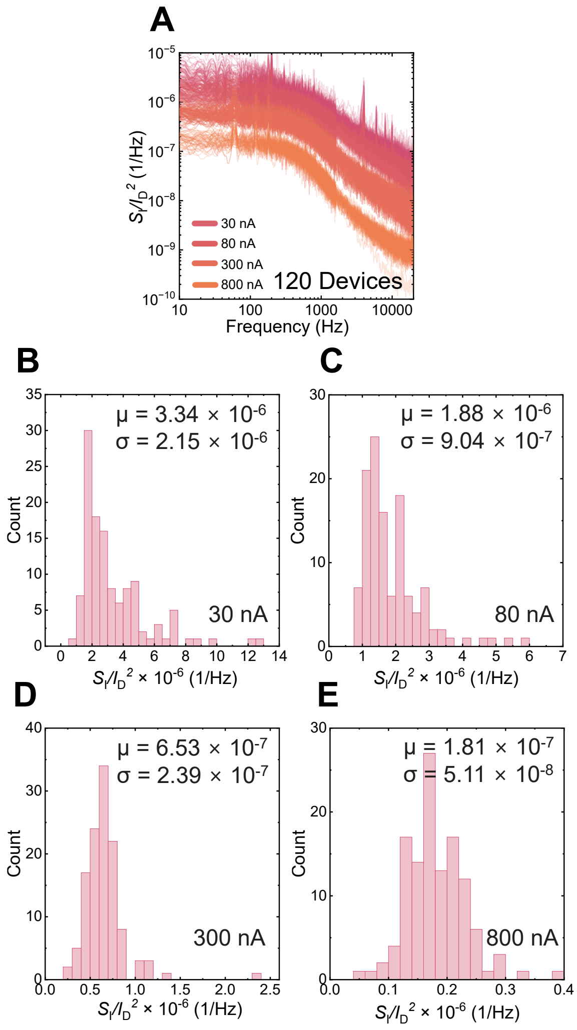

Fig. S8. Statistical evaluation of PSD characteristics across 120 FD-SOI devices. (**A**) Current normalized PSD characteristics of 120 FD-SOI devices measured at four I_D levels (30, 80, 300, and 800 nA). (**B** to **E**) Statistical distributions of the extracted PSD values at 10 Hz for I_D level: (B) 30 nA, (**C**) 80 nA, (**D**) 300 nA, and (**E**) 800 nA.

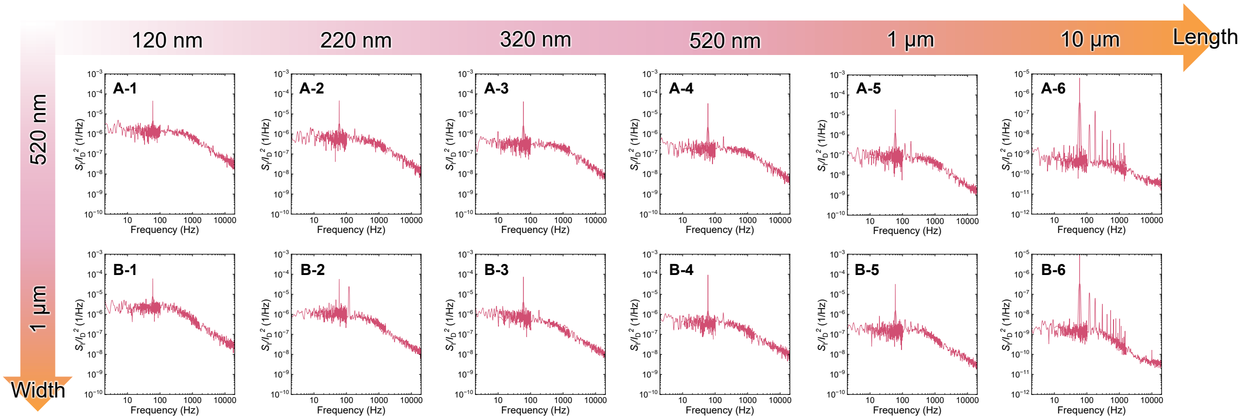

Fig. S9. Geometry independence of G–R noise characteristics. Current normalized PSD measured at I_D = 200 nA for FD-SOI devices with varying channel lengths (120 nm to 10 μm) widths of (**A-1** to **A-6**) 520 nm and (**B-1** to **B-6**) 1 μm.

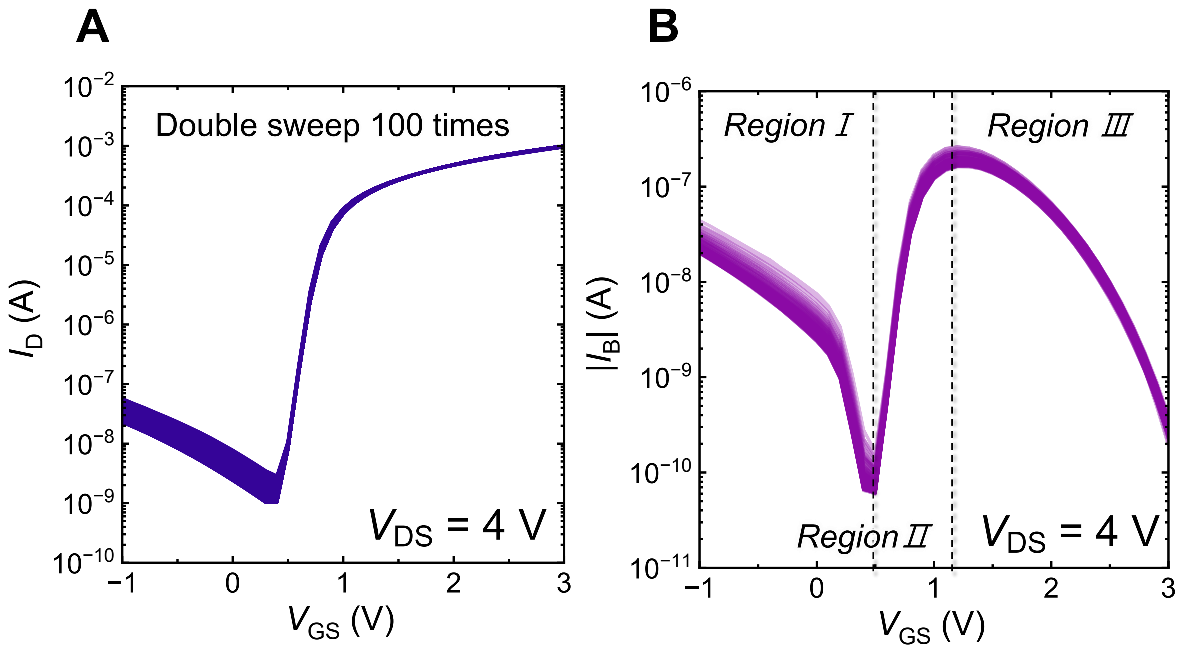

Fig. S10. Transfer and body current characteristics with 200 double-sweep cycles. (**A**) I_D-V_{GS} and (**B**) $|I_B|$-V_{GS} characteristics were measured after 200 double-sweep cycles between gate voltages of –1 V and 3 V at a fixed drain voltage of 4 V. The results show non-hysteresis and excellent repeatability in both the drain and body currents, demonstrating the robust electrical stability of the FD-SOI device under repetitive bias stress. The NDR behavior in the body current remained well-preserved even after extensive cycling.

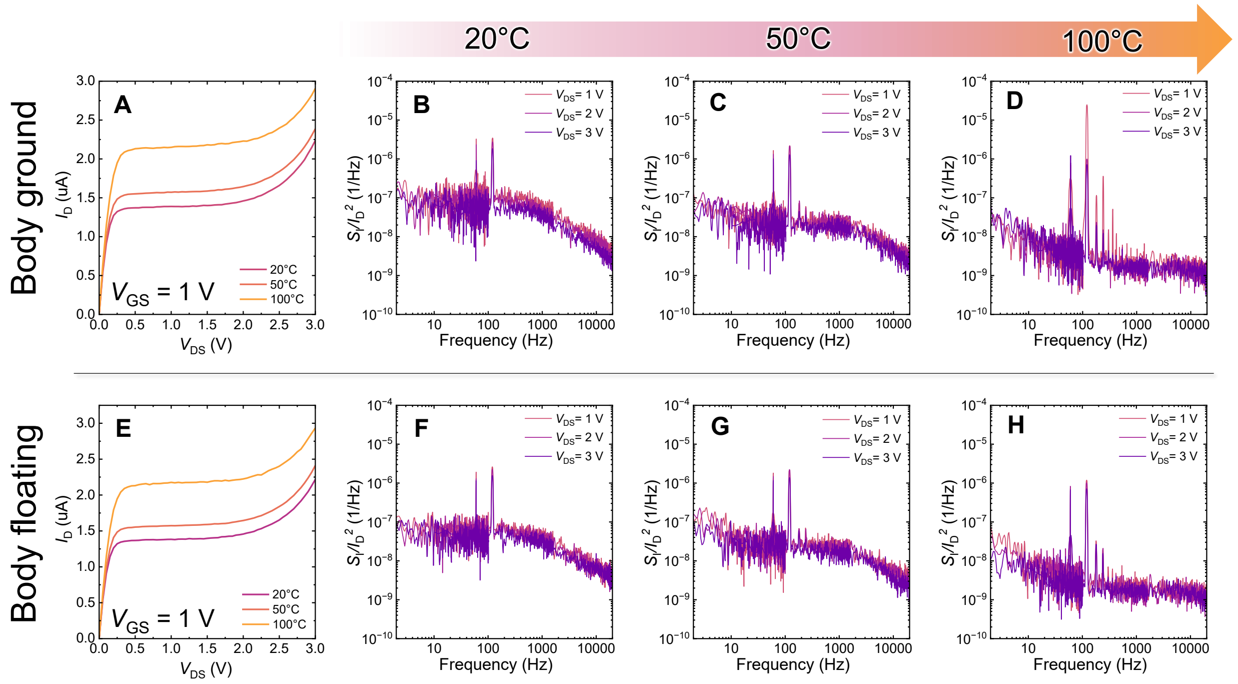

Fig. S11. Temperature-dependent LFN behavior under floating and grounded body conditions as a function of V_{DS}. Drain current and LFN characteristics were measured under both floating-body and grounded-body conditions for an FD-SOI device at temperatures of 25°C, 50°C, and 100°C. I_D-V_{DS} were obtained at a fixed gate voltage of 1 V across the temperature range, along with corresponding LFN spectra. The measurements show that the output behavior and LFN characteristics are identical between (**A** to **D**) ground and (**E** to **H**) floating body conditions across all temperatures and drain biases. These results demonstrate that the FD-SOI device exhibits strong immunity to floating-body effects, maintaining stable current modulation and noise characteristics regardless of body ground.

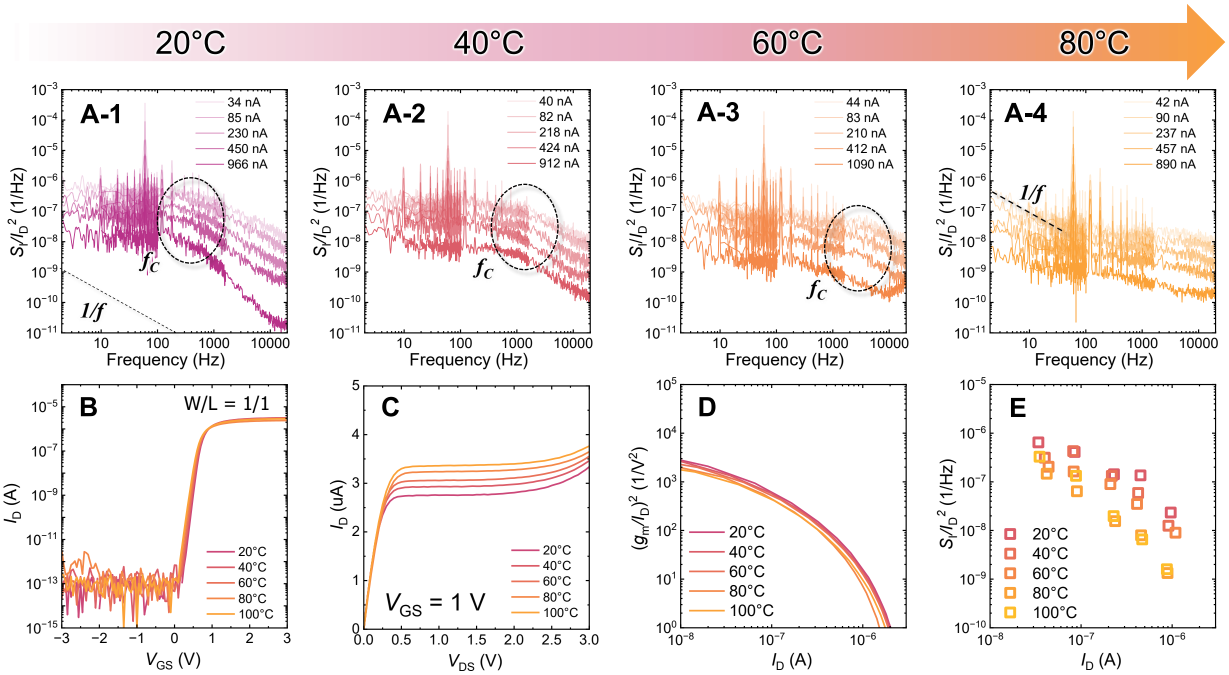

Fig. S12. Transfer, output, and LFN characteristic of a 1/1 µm FD-SOI device under varying bias and temperature. Temperature dependence (20°C, 40°C, 60°C, 80°C) of (**A-1** to **A-4)** drain current normalized PSD, (**B**) Transfer (V_{DS} = 0.1 V), (**C**) output characteristics (V_{GS}=1 V), (**D**) $(g_m/I_D)^2$ as a function of drain current and (**E**) PSD at 10 Hz extracted across drain current levels.

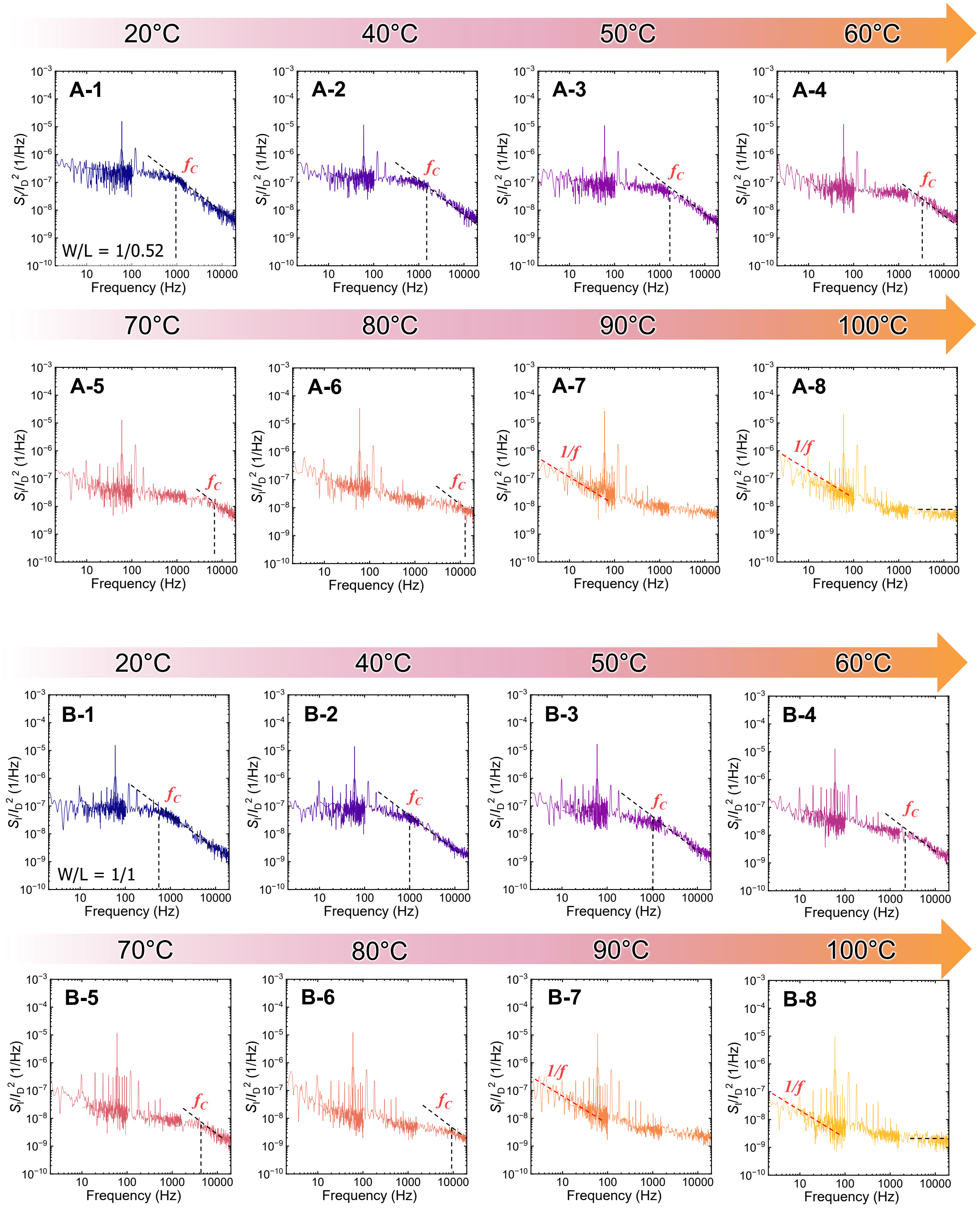

Fig. S13. Temperature dependence of LFN characteristics with varying channel length. LFN characteristics were measured at drain current of 200 nA for FD-SOI devices with channel lengths of (**A-1** to **A-8**) 0.52 μm and (**B-1** to **B-8**) 1 μm across a range of temperatures.

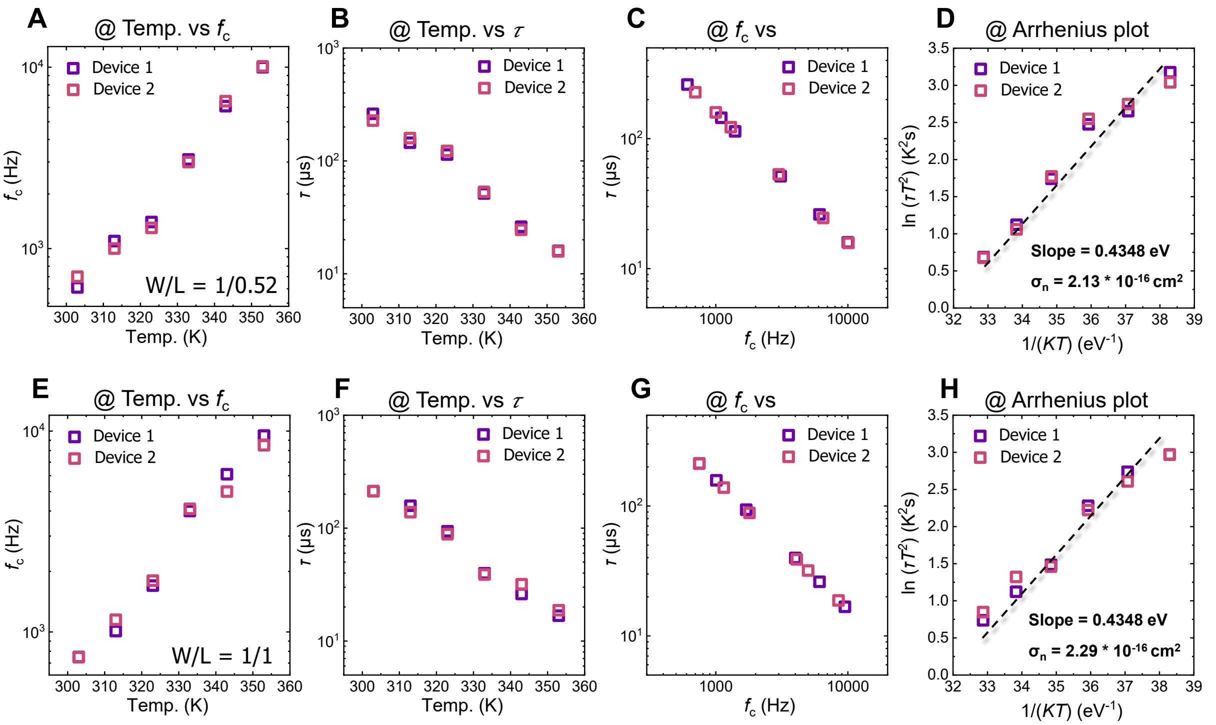

Fig. S14. Extraction of trap activation energy and capture cross section via Arrhenius analysis of temperature-dependent corner frequency. (**A**) Variation of f_c as a function of temperature. (**B**) Variation of τ as a function of temperature. (**C**) Inverse relationship between f_c and τ. (**D**) An Arrhenius plot of τ versus $1/kT$ yielded a linear behavior, from which an activation energy of 0.4348 eV was extracted. Additionally, the capture cross section of the trap was estimated to be 2.29×10^{-18} cm². **A** to **D** correspond to a device with W/L=1/0.52 µm, while **E** to **F** show results for a device with W/L=1/1 µm. Identical values of E_T and capture cross section (σ) were extracted regardless of the channel length, confirming geometry independence.

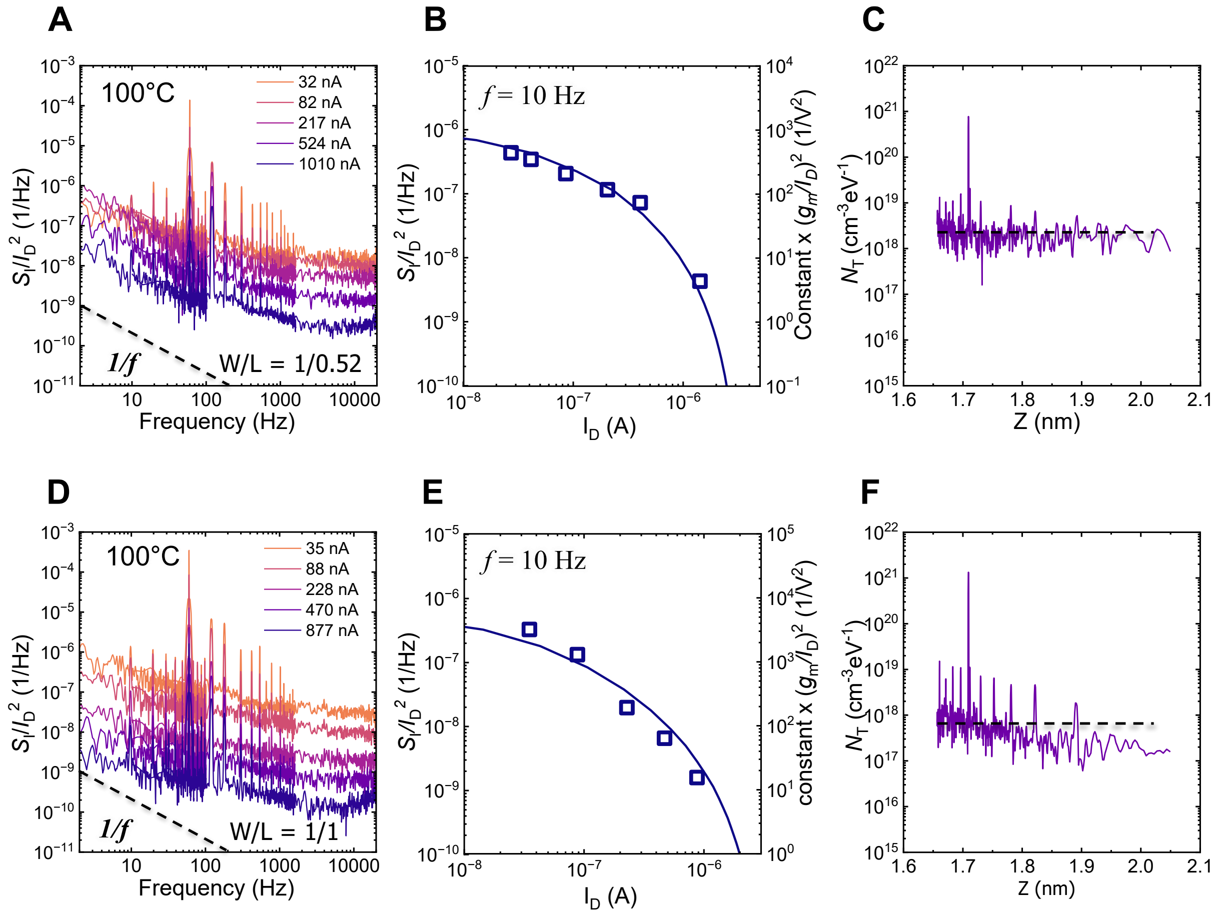

Fig. S15. Trap profiling using CNF noise at high temperatures with varying channel length. LFN measurements were conducted at 100°C for FD-SOI devices with channel lengths of (**A** to **C**) 0.52 µm and (**D** to **E**) 1 µm. The PSD near 10 Hz exhibited a clear 1/*f* behavior across both devices, indicating that CNF noise dominates under high-temperature conditions (fig. S8, A and D). The extracted PSD spectra was fitted using the CNF noise model, validating the interpretation of trap-assisted carrier fluctuations as the primary noise source (**B** and **E**). From the fitting results, the trap volume density was extracted for each device, demonstrating consistent trap characteristics independent of channel scaling (**C** and **F**).

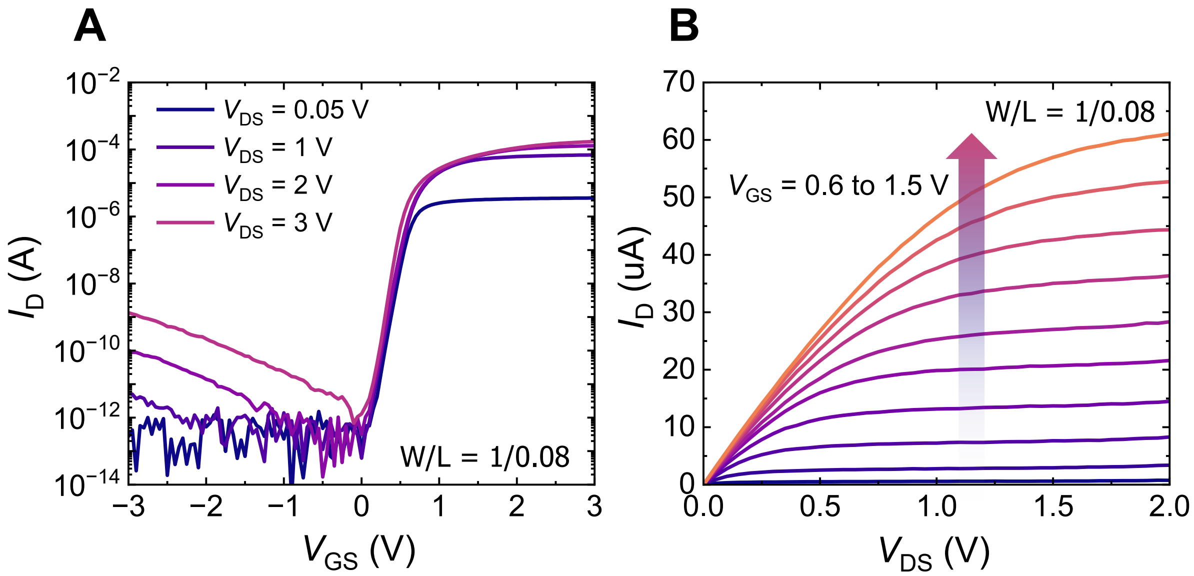

Fig. S16. Transfer and output characteristics of an 80 nm FD-SOI transistor. (**A**) Transfer and (**B**) output characteristics of an FD-SOI device with W/L=1/0.08 μm, measured across different V_{DS} and V_{GS} conditions.

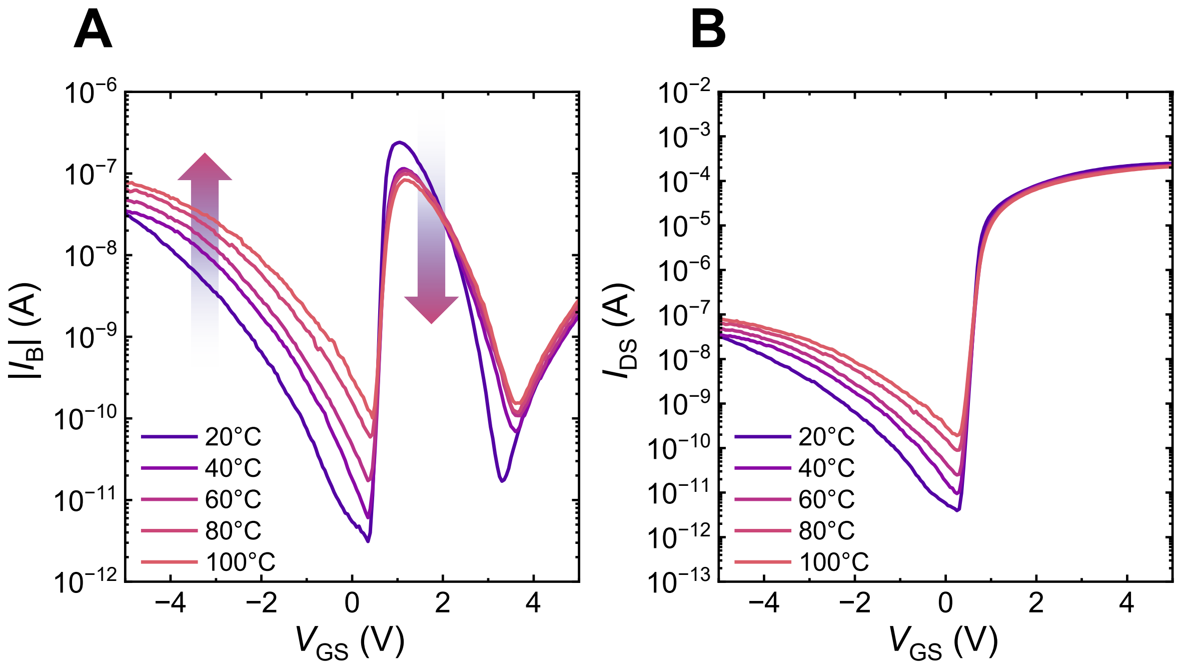

Fig. S17. Temperature dependence of drain and body currents at V_D=4 V. (**A**) I_D-V_{GS} and (**B**) $|I_B|$-V_{GS} characteristics were measured at a fixed V_{DS} of 4 V across temperatures of 25°C, 40°C, 60°C, 80°C, and 100°C.

As the temperature increased, a noticeable rise in the GIDL current was observed. This behavior is consistent with the thermally assisted band-to-band tunneling process, which can be modeled by:

$$I_{GIDL} = E_{OX}{}^2 exp\left(-\frac{B}{E_{OX}}\right)$$

where E_{ox} is the electric field across oxide and B is a parameter dependent on the bandgap and effective mass (63). Higher temperatures effectively reduce the bandgap, enhancing tunneling rates and leading to an increase in GIDL current. As the temperature increases, enhanced phonon scattering further degrades carrier mobility, especially in the strong inversion regime. This mobility reduction weakens the lateral electric field and lowers carrier energy, thereby suppressing impact ionization. Consequently, I_{NDR_peak} decreases with increasing temperature and gate voltage beyond the threshold for strong inversion. The concurrent increase in GIDL current and decrease in I_{NDR_peak} with rising temperature led to a degradation in the PVR of the NDR characteristic. These trends highlight the trade-off between thermal activation effects on leakage mechanisms and impact ionization efficiency in FD-SOI devices operating under high V_{DS}.

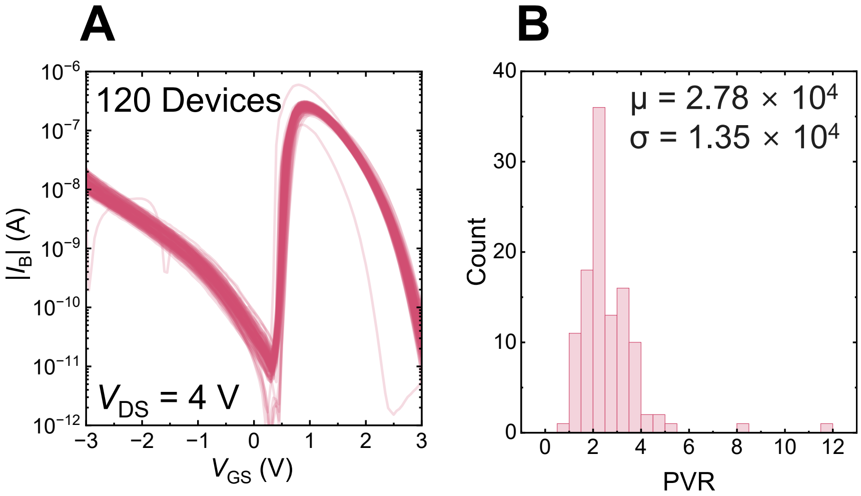

Fig. S18. Statistical evaluation of NDR characteristics across 120 FD-SOI devices. (**A**) $|I_B|$ characteristics of 120 FD-SOI devices measured at $V_{DS} = 4$ V. (**B**) Statistical distribution of the PVR extracted from the NDR characteristics, showing a mean value of 2.78×10^4 and a standard deviation of 1.35×10^4.

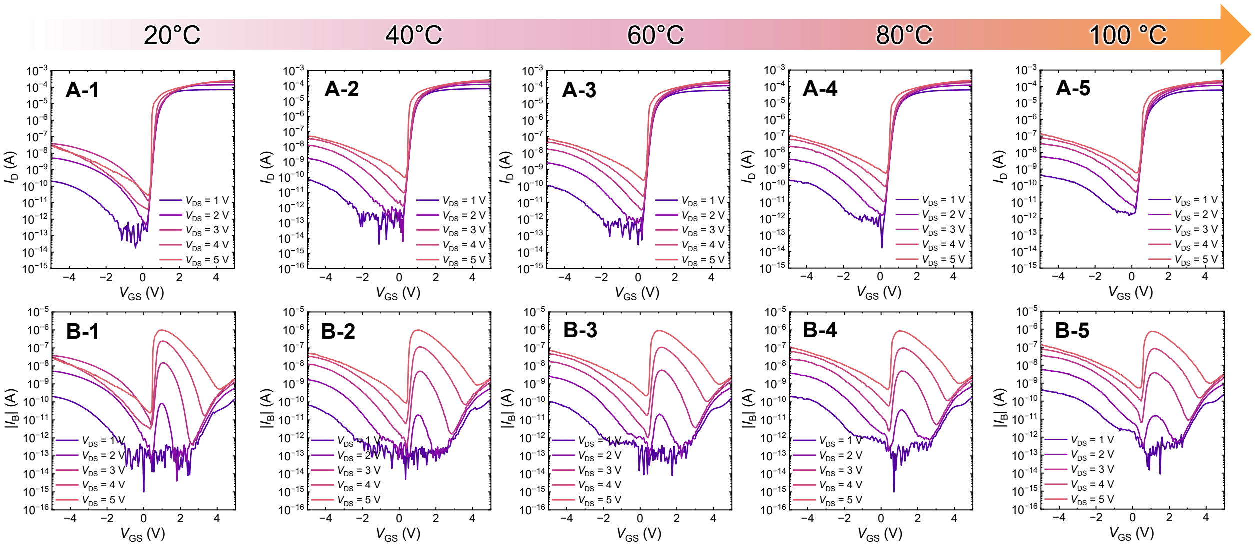

Fig. S19. Temperature dependence of body current and transfer characteristics. (**A-1**to **A-5**) I_D-V_{GS} and (**B-1** to **B-5**) $|I_B|$-V_{GS} characteristics were measured for an FD-SOI device under varying temperatures of 25°C, 4 °C, 60°C, 80°C, and 100°C. The measurements were performed at drain voltages ranging from 1 V to 5 V.

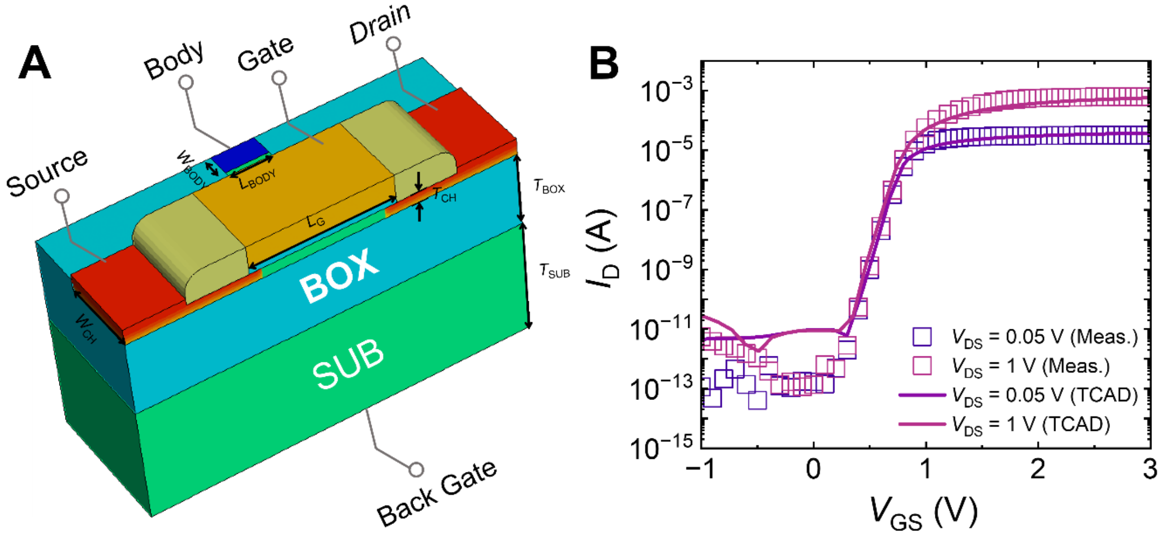

Fig. S20. FD-SOI calibration of Synopsys Sentaurus TCAD simulation. (**A**) Device structure used for TCAD simulation of the FD-SOI transistor. (**B**) Transfer characteristics extracted from both fabricated FD-SOI devices and TCAD simulations. The device structural parameters are detailed in Supplementary Table 5.

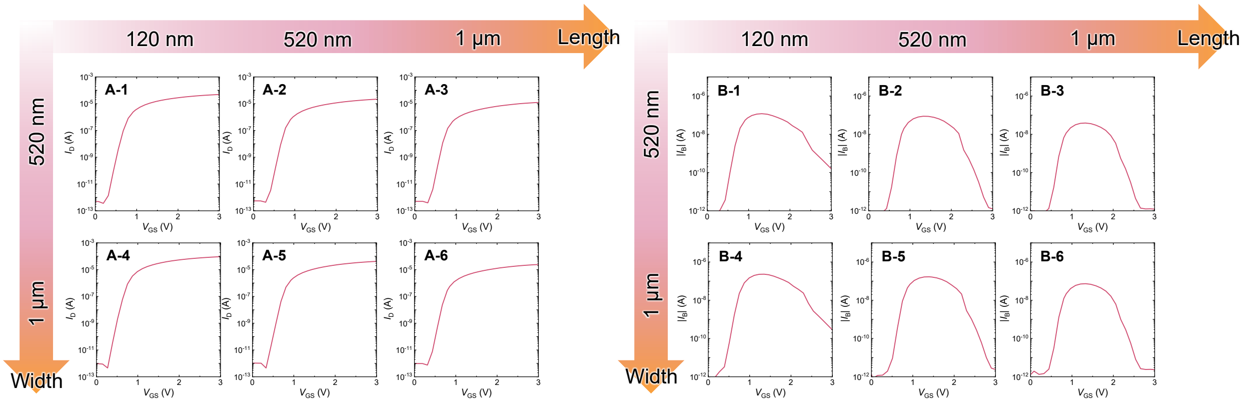

Fig. S21. TCAD-simulated transfer and body current characteristics under varying device geometries. (**A-1** to **A-6**) I_D–V_{GS} and (**B-1** to **B-6**) $|I_B|$–V_{GS} were simulated for FD-SOI devices with different channel lengths (120 nm, 520 nm, and 1 μm) and widths (520 nm and 1 μm).

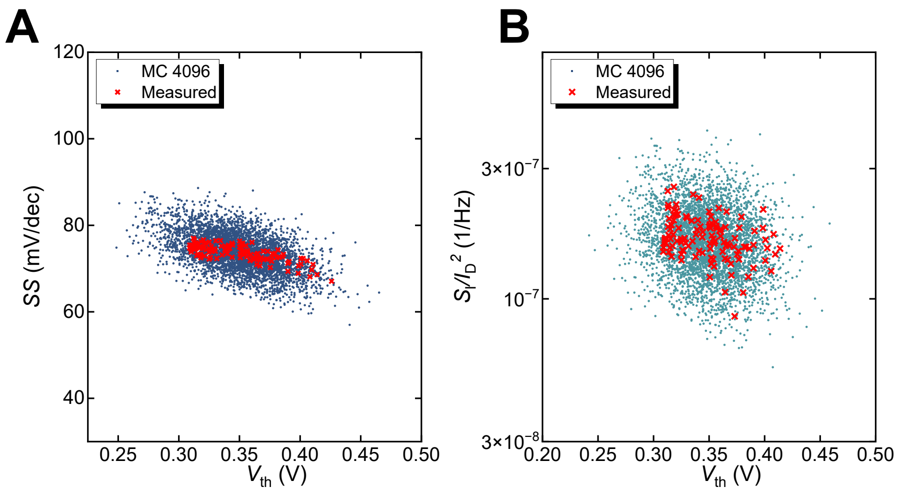

Fig. S22. Scatter overlays comparing measured devices (red crosses) and 4096 correlation-aware Monte Carlo samples (dots). (**A**) V_{th}–SS scatter distribution, showing that the MC ensemble reproduces both the spread and the correlation tilt observed in the measured dataset. (**B**) V_{th}–PSD scatter distribution, plotted with PSD on a logarithmic scale. The MC ensemble accurately reflects the joint variation observed experimentally.

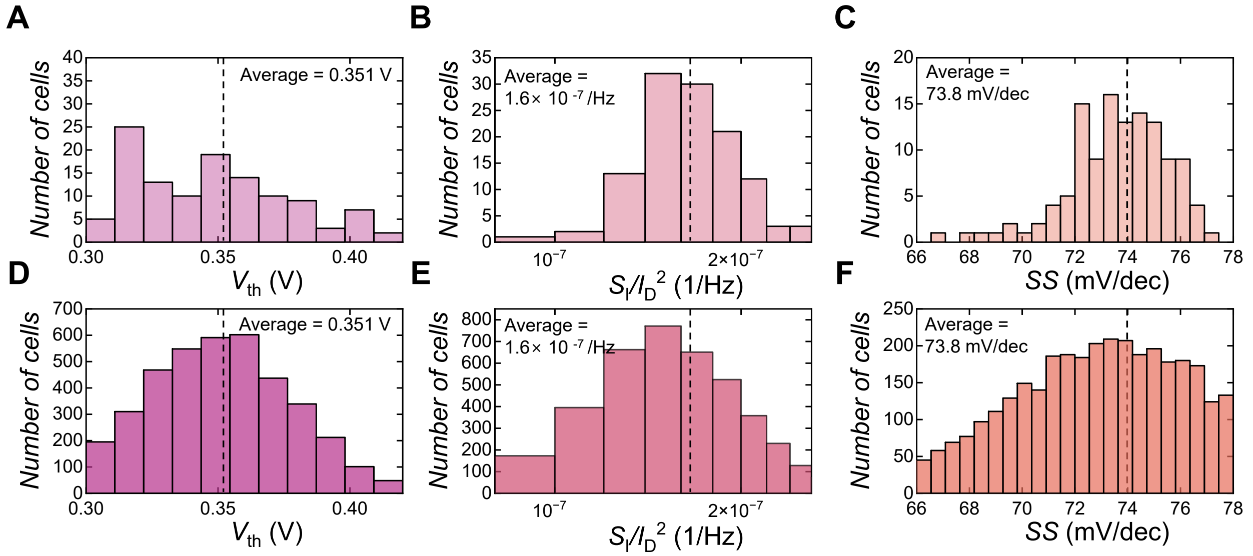

Fig. S23. Distribution comparisons of measured devices and correlation-aware Monte Carlo ensembles. (**A**) Histogram of measured V_{th}. (**B**) Histogram of measured $\log_{10}$(PSD). (**C**) Histogram of measured SS. (**D**) Histogram of V_{th} generated by correlation-aware Monte Carlo (4096 samples). (**E**) Histogram of $\log_{10}$(PSD) generated by correlation-aware Monte Carlo. (**F**) Histogram of SS generated by correlation-aware Monte Carlo.

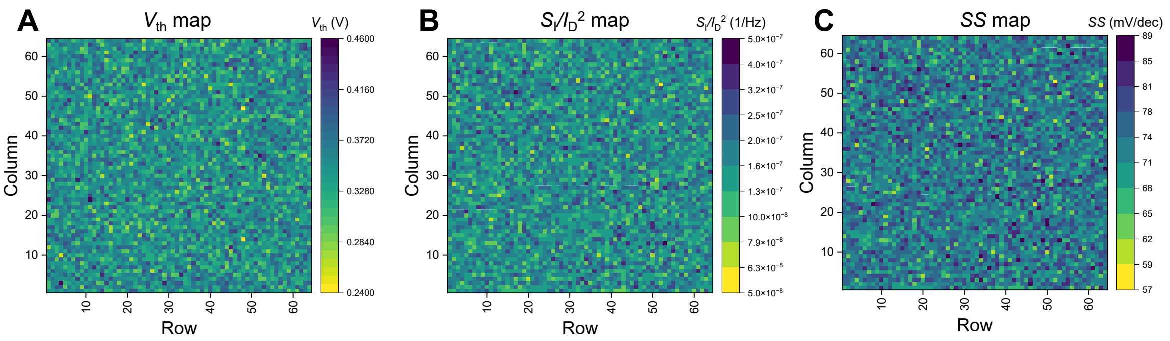

Fig. S24. 64 × 64 pseudo-array visualizations generated by correlation-aware Monte Carlo expansion. (**A**) Heatmap of V_{th}. (**B**) Heatmap of PSD (logarithmic scale). (**C**) Heatmap of SS. These images demonstrate how measured variability in device parameters propagates spatially when scaled to array-level implementations.

Table S1. Benchmarking table of applications utilizing nonlinearity in CMOS-based devices.
This benchmarking table summarizes various studies that leverage the nonlinear characteristics of CMOS devices (*12, 64–72*). Each case is compared in terms of the device type used, the specific nonlinear phenomenon exploited, the application domain.

Device	$L_{channel}$ (μm)	Dielectric	Non-linearity	Application	Refs.
Standard bulk MOSFET	0.18	SiO_2	Punch-through + Impact ionization + Floating Bulk	Synaptic and neural behaviours	[12]
PD-SOI MOSFET	0.1	HfO_2 + SiO_2	Floating body effect by impact ionization	Spiking Neural Network (SNN)	[64]
PD-SOI MOSFET	-	-	Band-to-band tunneling (BTBT)	Izhikevich neuron model as a spiking neuron circuit	[65]
PD-SOI MOSFET	0.032	HfO_2 + SiO_2	Band-to-band tunneling (BTBT)	SNN and LIF	[66]
PD-SOI MOSFET	0.105	HfO_2 + SiO_2	Quantum tunneling + Band-to-band tunneling (BTBT)	SNN and LIF	[67]
Biristor (CMOS based)	0.25	-	Punch-through + Single-transistor latch (STL)	Probabilistic computing	[68]
SONOS MOSFET	0.88	SONOS (Silicon-Oxide-Nitride-Oxide-Silicon)	Impact ionization and Single-transistor latch (STL)	Neuromorphic computing (Simultaneous implementation of neurons and synapses)	[69]
Cryptoristor (CMOS based)	0.3	-	Impact ionization + Floating body (FB) + Single-transistor latch (STL)	True random number generator (TRNG)	[70]
FeFET	1	HfO_2 + SiO_2	Gate-induced drain leakage (GIDL)	Frequency doubler	[71]
Standard bulk MOSFET	-	-	Thermal noise, Shot noise, 1/f noise	True random number generator (TRNG) and Physically unclonable function (PUF)	[72]
This work (FD-SOI)	1/ 0.52/ 0.08	SiO_2	Body current NDR and G-R noise	Image processing	

Table S2. Volume trap density benchmarking: bulk CMOS vs PD-SOI vs FD-SOI.

The N_T of bulk CMOS (*23, 73–76*), partially depleted SOI (PD-SOI) (*24–26, 77–81*), and fully depleted SOI (FD-SOI) transistors (*20, 27–29, 48, 82–84*) were benchmarked to assess the impact of device architecture on trap-related reliability and noise performance. Bulk CMOS devices typically exhibit higher trap densities due to the presence of bulk charge defects and less effective electrostatic control. PD-SOI structures partially mitigate bulk effects but still suffer from floating body and interface-related traps. In contrast, FD-SOI devices demonstrate the lowest volume trap densities among the compared technologies, attributed to their excellent electrostatic integrity, minimized body effects, volume inversion and superior interface quality.

Devices	Channel	$L_{channel}$ (μm)	$T_{channel}$ (nm)	Dielectric	Noise model	Dielectric trap density ($/cm^3/eV$)	BOX trap density ($/cm^3/eV$)	Refs.
Bulk	Single crystal Si	0.25	–	HfO_2, HfSiO	CNF	(1 nm SiO_2 + 7 nm HfO_2) 8×10^{20} (1 nm SiO_2 + 3 nm HfO_2) 8×10^{19}	-	[23]
	Single crystal Si	0.09	30	SiO_2, SiON	CNF/ CMF	2×10^{17}	-	[73]
	Single crystal Si	0.1	58	SiO_2	RTN + CNF	$5 \times 10^{16} – 4.2 \times 10^{18}$	-	[74]
	Single crystal Si	0.12 – 0.35	–	SiO_2	CNF + HMF + RTS	–	-	[75]
	Single crystal Si	0.13	–	SiO_2	CNF + CMF	$3 \times 10^{17} – 4 \times 10^{17}$	-	[76]
PD-SOI	Single crystal Si	0.6 – 1	100	SiO_2	Kink-related excess noise	-	-	[77]
	Single crystal Si	0.12 – 0.13	100	SiO_2	CNF + Kink-related excess noise	$2 \times 10^{18} – 7 \times 10^{18}$	-	[24]
	Single crystal Si	0.8	100	SiO_2	CNF + Kink-related excess noise	-	-	[78]
	Single crystal Si	0.1	< 20	SiO_2	RTS + CNF (Wide channel), CMF	-	-	[79]
	Single crystal Si	0.13 – 0.32	160	SiON	CNF + Kink-related Lorentzian noise	$6 \times 10^{17} – 10^{18}$	-	[25]
	Heterojunction Si/SiGe	0.5	–	SiO_2	CNF	10^{18}	-	[26]
	Single crystal Si	0.04	–	SiO_2	CNF + Lorentzian component	$\sim 10^{19}$	-	[80]
	Single crystal Si	0.04 (Thin oxide), 0.112 (Thick oxide)	160	SiO_2	CNF (Thin oxide), CMF (Thick oxide)	$6 \times 10^{17} – 5 \times 10^{18}$	-	[81]
FD-SOI	Single crystal SOI/SiGeOI N/P channel	0.024 (28 nm node), 0.02 (14 nm node)	6	High-k	CNF + RTN + Novel RTN log (S_{VG})	4×10^{17}	2×10^{17}	[27]
	Single crystal Si	0.03 – 0.9	7	High-k	CNF + CMF + Lorentzian (@ small device)	$7.6 \times 10^{17} – 1.6 \times 10^{18}$	-	[28]
	Single crystal Si	0.014 – 1	8.7	High-k	CNF/ CMF(front & back gate interfaces)	8.7×10^{17}	3.5×10^{17}	[82]
	Single crystal Si	0.03	7	High-k	CNF/ CMF + RTN	-	-	[29]
	Single crystal Si	1	7	High-k	CNF/ CMF	9×10^{17}	2×10^{17}	[48]
	Single crystal Si	0.1 – 0.25	10 – 18	SiO_2	CMF	6×10^{17} – a few 10^{18}	-	[83]
	Single crystal Si	0.05, 0.1	6	SiO_2	CNF + CMF + Coulomb scattering	3×10^{17}	4×10^{17}	[84]
	Single crystal Si	0.02	6	High-k	CNF + RTN	2.5×10^{17}	-	[20]
This work (FD-SOI)	Single crystal Si	1/ 0.52/ 0.08	9	SiO_2	CNF + G-R	2×10^{18}	-	

Table S3. Volume trap density benchmarking: a-IGZO vs Poly-Si vs 2D channel device.

The N_T of amorphous indium gallium zinc oxide (a-IGZO) thin-film transistors (*30–36*), polycrystalline silicon (Poly-Si) devices (*85–90*), and two-dimensional (2D) channel materials (*37–43, 91–94*) were benchmarked to compare the impact of material systems on defect density and noise performance. a-IGZO devices exhibit the highest trap densities due to the inherent disorder and the presence of localized defect states within the amorphous oxide matrix. Poly-Si transistors show moderate trap densities, mainly attributed to grain boundary defects arising from the polycrystalline nature of the channel. In contrast, 2D channel devices, such as MoS_2 and WSe_2 transistors, demonstrate relatively lower trap densities, benefiting from atomically flat surfaces and the absence of dangling bonds, though still affected by interface quality and environmental sensitivity.

In comparison, CMOS-based silicon MOSFETs exhibit the lowest trap densities among all considered technologies, owing to decades of material and interface engineering optimization. This highlights the advantage of mature silicon processes in achieving superior electrical stability and minimal defect-related noise.

Channel	$L_{channel}$ (µm)	$T_{channel}$ (nm)	Dielectric	Noise model	Trap density (/cm^3/eV)	Refs.
a-IGZO	120	25	SiO_2	CNF	1.2×10^{20}	[30]
	30	50	SiO_2	CNF	2×10^{18}	[32]
	20	-	SiO_2	CNF	4.4×10^{19}	[33]
	40	40	SiO_2	CNF	1.1×10^{19}	[34]
	50	20	SiO_2	CNF	7.3×10^{20}	[31]
	10	50	SiO_2	CNF	2.3×10^{20}	[35]
	50	50	SiO_2	CNF	6×10^{21}	[36]
Poly-Si	4-20	50	SiO_2	CNF	5×10^{17}	[85]
	2-20	-	SiO_2	CNF + CMF	-	[86]
	10	150	SiO_2	1/f + Lorentzian	p-channel: 2.7×10^{19}<br>n-channel: 6.7×10^{18}	[87]
	8-20	100	SiO_2	CNF + CMF	9×10^{18}	[88]
	5-50	0.03	HfO_2/ ZrO_2	BHF	-	[89]
	5-50	0.04	SiO2/ HZO	CNF	$2.1 \times 10^{18} – 1.2 \times 10^{19}$	[90]
2D	0.1<br>0.2<br>0.3	-	Al_2O_3	CNF + CMF	5.5×10^{18}<br>2.1×10^{19}<br>8×10^{19}	[37]
	20<br>50<br>100	-	Electrolyte gate<br>(Solution-gated)	CNF + CMF + RTS	2.2×10^{19}<br>4.93×10^{19}<br>1.74×10^{20}	[91]
	1.3 –<br>3.5	-	SiO_2	CNF	As-fabricated: 2×10^{19}<br>Aged: 2.5×10^{20}	[38]
	0.28	11.3	SiO_2 (Back)<br>Al_2O_3 (Top passivation)	CNF + HMF	Without Al_2O_3: 7.2×10^{10} (cm^{-2}eV^{-1})<br>With Al_2O_3: 5.5×10^{10} (cm^{-2}eV^{-1})	[92]
	1.3–<br>6	Thin: 2 - 3 layers (~ 2)<br>Thick: 15 - 18 layers (~ 11)	SiO_2	CNF	10^{18} (cm^{-2}eV^{-1})<br>Interface: $(5.5 – 7.2) \times 10^{10}$ (cm^{-2}eV^{-1})	[93]
	1.71	0.65 (Single layer)	SiO_2	HMF + G-R noise	-	[94]
	5.1		HfO_2	CNF	2.64×10^{19}	[39]
	1		Al_2O_3/ HfO_2	CNF	1.12×10^{20}	[40]
	1		SiO_2	CNF	7.47×10^{19}	[41]
	1		Hbn	CNF	1.24×10^{20}	[42]
	3.5		Hbn	CNF	2.82×10^{20}	[43]
This work<br>(FD-SOI)	1/ 0.52/ 0.08	9	SiO_2	CNF + G-R	2×10^{18}	

Table S4. Activation energy and capture cross section benchmarking between previous CMOS and this work. This table compares the activation energy positions and capture cross sections of silicon deep-level defects responsible for G–R noise, extracted from previous CMOS studies and from this work (*44–49, 95–97*). Benchmarking was conducted for devices where G–R noise dominates the LFN behavior.

Device	Channel	$L_{channel}$ (μm)	$T_{channel}$ (nm)	Dielectric	E_T (eV)	σ (capture cross section,cm^2)	Trap density ($/cm^3/eV$)	Refs.
Mosfet	Single crystal Si	8		SiO_2	$E_T - E_V = 0.42$	3.2×10^{-14}	$4.8 – 8.4 \times 10^{15}$	[44]
GAA nanowire FET	Si/SiGe superlattice	0.25		HfSiO+SiO_2	$E_C - E_T = 0.45$	1.6×10^{-17}	-	[45]
FD-SOI MOSFET (standard)	Single crystal Si	0.105	20	SiO_2	$E_C - E_T = 0.15$<br>$E_C - E_T = 0.13$	5.8×10^{-24} (Trap1)<br>5.8×10^{-21} (Trap2)	5×10^{13} (Trap1)<br>1×10^{14} (Trap2)	[95]
FD-SOI MOSFET (extension-less)	Single crystal Si	0.105	20	SiO_2	$E_C - E_T = 0.24$<br>$E_C - E_T = 0.16$	1×10^{-21} (Trap1)<br>4.4×10^{-14} (Trap1 hole)<br>2.5×10^{-19} (Trap2)<br>1.4×10^{-16} (Trap2 hole)	5×10^{16} (Trap1)<br>1×10^{17} (Trap2)	[96]
FD-SOI MOSFET	Single crystal Si	0.105	6	SiO_2	$E_C - E_T$=0.453 (H)<br>$E_C - E_T$=0.42 (V_2)	1.47×10^{-17}<br>9×10^{-16}	-	[46]
N-MOSFET	Single crystal Si	2		SiO_2	$E_C - E_T = 0.53$	2.8×10^{-15}	10^{13}	[47]
PD-SOI MOSFET	Single crystal Si	1	100	Nitrided gate Oxide (SiON)	$E_C - E_T = 0.43$ (V-O)<br>$E_C - E_T = 0.48$ (Hydrogen2)<br>$E_C - E_T = 0.31$ (Hydrogen1)<br>$E_C - E_T = 0.24$ (V2 (0/-))<br>$E_C - E_T = 0.14$ (V2-/--)<br>$E_C - E_T = 0.17$ (BiOi)	0.88×10^{-15}<br>0.16×10^{-12}<br>0.16×10^{-14}<br>0.73×10^{-14}<br>0.63×10^{-15}<br>0.12×10^{-14}	$10^{15} – 10^{16}$<br>$10^{15} – 2 \times 10^{16}$<br>$2 \times 10^{15} – 2 \times 10^{16}$<br>$3 \times 10^{15} – 4 \times 10^{16}$<br>$2 \times 10^{15} – 2 \times 10^{16}$<br>$3 \times 10^{15} – 9 \times 10^{16}$	[97]
FD-SOI MOSFET	Single crystal Si	0.085	6	SiO_2	$E_C - E_T = 0.20$ (V2 (+/0))<br>$E_C - E_T = 0.23$ (V2-/--)<br>$E_C - E_T = 0.26$ (BiOi)<br>$E_C - E_T = 0.3$ (BiBs)<br>$E_C - E_T = 0.35$ (CiOi)<br>$E_C - E_T = 0.44$ (Vp)<br>$E_C - E_T = 0.53$ (D1)<br>$E_C - E_T$= 0.27 (D2)	10^{-15}<br>$10^{-15} – 10^{-16}$<br>$10^{-13} – 10^{-14}$<br>3×10^{-16}<br>10^{-16}<br>$10^{-14} – 10^{-15}$<br>1.5×10^{-14}<br>4.8×10^{-16}	-	[48]
N-channel FINFET	Single crystal Si	0.91		HfSiON + SiO_2	$E_C - E_T = 0.23$ (V2-/--)<br>$E_C - E_T = 0.26$ (BiOi)<br>$E_C - E_T = 0.38$ (CiPs(0/-))<br>$E_C - E_T = 0.3$ (D5)<br>$E_C - E_T = 0.35$ (D7)<br>$E_C - E_T = 0.44$ (D8)<br>$E_C - E_T = 0.53$ (D9)<br>$E_C - E_T = 0.27$ (D10)	$10^{-15} – 10^{-16}$<br>$10^{-13} – 10^{-14}$<br>10^{-15}<br>1.8×10^{-19}<br>1.8×10^{-17}<br>7.9×10^{-18}<br>2.1×10^{-18}<br>1.8×10^{-15}	2.7×10^{18}	[49]
This work (FD-SOI)	Single crystal Si	1/ 0.52/ 0.08	9	SiO_2	$E_C - E_T = 0.4348$	2.13×10^{-16}	-	

Table S5. Physical device parameters used in simulation. Device structural parameters used in the TCAD simulation of the FD-SOI transistor.

	Parameter	Description	Value	Unit
Device geometry	W_{CH}	Channel width	10	μm
	L_{CH}	Channel length	120	nm
	T_{CH}	Channel thickness	9	
	L_{SD}	Source/Drain length	100	
	L_{SPACER}	Spacer length	50	
	T_{GI}	Gate insulator thickness	5	
	T_{BOX}	BOX thickness	65	
	T_{SUB}	Substrate thickness	100	
	L_{BODY}	p+ body length	36	
	W_{BODY}	p+ body width	8	μm
	N_{SD}	Source/Drain doping concentration	10^{20}	cm^{-3}
	N_{CH}	Channel doping concentration	undoped	
	N_{POLY}	PolySi doping concentration	10^{21}	

Supplementary Note 1. Mechanism of body current.

To further elucidate the origin of the NDR behavior observed in the body current, we classify the operating regimes into three distinct regions based on gate voltage and the associated carrier transport mechanisms.

In Region I, the body current is dominated by GIDL. This current originates from band-to-band tunneling (BTBT) in the drain-to-body overlap region, where a strong vertical electric field at high drain voltage facilitates the tunneling of electrons from the valence band of the body into the conduction band of the drain. This mechanism is independent of channel formation and is most prominent under low gate bias conditions where the gate-induced field at the drain junction remains high.

In Region II, corresponding to the SS region of the transfer characteristic, the body current increases sharply with gate voltage. In this regime, the channel is not yet in strong inversion, resulting in a relatively high channel resistance. Consequently, a substantial portion of the drain voltage drops across the channel. This enhances the lateral electric field, enabling energetic electrons to gain sufficient energy for impact ionization near the drain-side of the channel. The resulting generation of electron–hole pairs contribute to a rise in body current, marking the onset of the NDR behavior.

In Region III, the device enters strong inversion, and the channel resistance significantly decreases. The reduced lateral field in the channel leads to a suppression of impact ionization events, and the body current begins to decrease despite increasing gate bias. This transition explains the negative differential resistance observed in the body current characteristic. Taken together, these three regimes illustrate a complex interplay between electric distribution, carrier transport, and impact ionization dynamics in FD-SOI devices. The distinct body current profiles observed in each regime offer valuable insight into the spatial and bias-dependent physics governing NDR characteristics.

Supplementary Note 2. Body current LFN characteristics.

The origin of the observed *1/f* noise in the body current under NDR conditions can be attributed to a combination of various noise sources. Specifically, the stochastic generation of carriers via impact ionization is followed by a broad distribution of recombination timescales, resulting in a spread of time constants that collectively manifest as $1/f$ noise. In addition to recombination-related fluctuations, the noise may also arise from scattering processes experienced by the carriers generated through impact ionization, as well as resistive fluctuations within the channel and body regions (*98, 99*). These combined mechanisms contribute to a complex noise spectrum, the observed $1/f$ behavior across the entire body current operating range.

Supplementary Note 3. Carrier number fluctuation noise.

The CNF model provides a widely accepted framework to interpret LFN behavior in scaled MOSFETs. In this model, fluctuations in the number of carriers arise from stochastic capture and emission of charge carriers at discrete trap sites located in the gate dielectric. Each trap acts as a localized potential well, modulating the flat band voltage (channel charge density) and giving rise to drain current noise with a characteristic 1/*f* spectral shape. The CNF model can be expressed as (*100*):

$$\frac{S_I}{{I_D}^2} = \left(\frac{g_m}{I_D}\right)^2 S_{Vfb} \quad \text{(S1)}$$

Where

$$S_{Vfb} = \frac{q^2 kT\lambda N_T}{fWL{C_{OX}}^2} \quad \text{(S2)}$$

where g_m is the transconductance, S_{Vfb} is the flat band voltage fluctuation, C_{OX} is the oxide capacitance per unit area, q is the electron charge, T is the temperature in Kelvin, λ is the oxide tunneling distance, N_T is the volume trap density, W and L represent the width and the length of the FET channel. According to the CNF model, the S_I/I_D^2 decreases with an increase in I_D following the behavior of g_m/I_D whose value is inversely proportional to SS.

Supplementary Note 4. Generation-recombination noise.

G–R noise arises from discrete generation and recombination events associated with deep-level defects in the silicon channel given by (*101*):

$$\frac{S_I}{{I_D}^2} = \frac{q^2 f_t(1-f_t)N_f}{LWC_{OX}} (\frac{g_m}{I_D})^2 \times \frac{4\tau}{1+4\pi^2\tau^2 f^2} \quad \text{(S3)}$$

where g_m is the transconductance, f_t is fractional occupancy of traps around the Fermi level and N_f is the volume trap density in the film, C_{OX} is the oxide capacitance per unit area, q is the electron charge, W and L represent the width and the length of the FET channel and τ is carrier time constant.

These defects act as energetic centers that intermittently generation and recombination, leading to fluctuations in the carrier density and, consequently, in the drain current. Unlike the CNF noise, G–R noise manifests as distinct Lorentzian spectra, each characterized by a specific corner frequency determined by the carrier's generation and recombination time constants. These time constants are highly sensitive to temperature, decreasing with thermal activation of the trap sites. The SRH theory for the corner frequency, given by (*46*, *102*):

$$\tau = \left(\frac{1}{2\pi f_c}\right) = \left(\frac{1}{c_n(n(x,y)+n_t)+c_p(p(x,y)+p_t)}\right) \quad \text{(S4)}$$

$$ln\,(\tau T^2) = \left(\frac{E_c-E_t}{KT}\right) + ln\,(\frac{h^3}{4k^2\sigma_n(6\pi^3 M_c {m_e}^{*1/2} {m_h}^{*3/2})^{1/2}}) \quad \text{(S5)}$$

where c_n and c_p represent the capture rates for electrons and holes, respectively, which are defined as the product of the thermal velocity and the capture cross section for electrons (σ_n) or holes (σ_p). The terms $n(x,y)$ and $p(x,y)$ denote the local free carrier concentrations at the trap position (x,y) within the depletion region of the transistor. In this context, h is Planck's constant; m_e and m_h are the effective masses of electrons and holes, respectively; and M_C refers to the number of conduction band minimum. By analyzing the slope and intercept of a plot of $\ln(\tau T^2)$ versus $1/kT$, one can determine the energy difference between the band edge and the trap level (i.e., $\Delta E = E_C - E_T$) and extract the electron capture cross section (σ_n) of the trap.

Supplementary Note 5. Correlation-aware Monte Carlo pseudo-array analysis.

In this Note, we describe in detail the procedure used to generate the pseudo-arrays presented in figs. S22 to S24 in this Supplementary Materials. The aim of this analysis is to demonstrate that the array-level demonstrations (28 × 28 and 64 × 64) are not based on idealized assumptions, but instead are firmly grounded in experimentally measured device statistics.

Specifically, we modeled the measured parameters using a multivariate normal distribution,

$$\mathrm{x} \sim N\ (\mu, \Sigma) \quad \text{(S6)}$$

where x is the parameter vector (e.g., $x = [V_{\mathrm{th}}, SS]^{\mathrm{T}}$ or $x = [V_{\mathrm{th}}, \log_{10}(\mathrm{PSD})]^{\mathrm{T}}$, μ is the mean vector estimated from the measured dataset, and Σ is the covariance matrix capturing both the variances of each parameter and their cross-correlations. From this distribution we generated ensembles of pseudo-devices (64 × 64 array-level) that reproduce the measured statistical behavior. This correlation-aware MC approach is widely adopted in semiconductor variability analysis and reliability modeling, as it ensures that pseudo-devices not only follow the correct marginal distributions but also preserve the correlation trends present in the real data (*103, 104*).

Fig. S22 shows scatter overlays comparing the measured devices with 4096 correlation-aware MC samples. fig. S22 (A) illustrates the V_{th}–SS distribution, where the MC ensemble (dots) accurately reproduces both the spread and the tilted elliptical shape observed in the measured dataset (red crosses). fig. S22 (B) shows the V_{th}–PSD relation plotted with PSD on a logarithmic y-axis, where the MC ensemble again follows the measured cluster, demonstrating that the covariance-based sampling correctly preserves the joint variability of the parameters.

Fig. S23 provides side-by-side histogram comparisons. Each parameter is split into two subpanels: (A), (B), and (C) show the measured histograms of V_{th}, $\log_{10}$(PSD) and SS, while (D), (E), and (F) show the corresponding distributions generated by correlation-aware MC sampling. The pseudo-devices closely match the measured distributions in mean, variance, and shape, and importantly, the distributions remain compact and do not scatter beyond the measured range. This confirms that the pseudo-arrays are faithful statistical extensions of the experimental dataset.

Fig. S24 visualizes the 64 × 64 pseudo-arrays generated from the correlation-aware Monte Carlo sampling. Panel (A) shows the 2D heatmap of V_{th}, panel (B) shows PSD (on a logarithmic scale), and panel (C) shows SS. Each pixel in these arrays corresponds to a pseudo-device sampled from the multivariate distribution defined above. These pseudo-arrays thus represent realistic projections of how D2D variability would manifest spatially at the array level.

Taken together, figs. S21 to S23 demonstrate that our pseudo-array results are grounded in a standard statistical methodology. By explicitly modeling the measured data as a multivariate normal distribution, the correlation-aware MC sampling preserves both marginal distributions and cross-correlations.

REFERENCES

1. M. M. Waldrop, The chips are down for Moore's law. *Nature* **530**, 144–147 (2016).

2. L. Forever, M. Lundstrom, Moore's law forever? *Science* **210**, 1–37 (2011).

3. M. Chhowalla, D. Jena, H. Zhang, Two-dimensional semiconductors for transistors. *Nat. Rev. Mater.* **1**, 16052 (2016).

4. R. Yang, H. Li, K. K. H. Smithe, T. R. Kim, K. Okabe, E. Pop, J. A. Fan, H. S. P. Wong, Ternary content-addressable memory with MoS_2 transistors for massively parallel data search. *Nat. Electron.* **2**, 108–114 (2019).

5. K. U. Demasius, A. Kirschen, S. Parkin, Energy-efficient memcapacitor devices for neuromorphic computing. *Nat. Electron.* **4**, 748–756 (2021).

6. D. Ielmini, H. S. P. Wong, In-memory computing with resistive switching devices. *Nat. Electron.* **1**, 333–343 (2018).

7. A. J. Yang, K. Han, K. Huang, C. Ye, W. Wen, R. Zhu, R. Zhu, J. Xu, T. Yu, P. Gao, Q. Xiong, X. Renshaw Wang, Van der Waals integration of high-κ perovskite oxides and two-dimensional semiconductors. *Nat. Electron.* **5**, 233–240 (2022).

8. Y. Wang, S. Sarkar, H. Yan, M. Chhowalla, Critical challenges in the development of electronics based on two-dimensional transition metal dichalcogenides. *Nat. Electron.* **7**, 638–645 (2024).

9. W. Xu, Y. Wu, S. Xi, Y. Wang, Y. Wang, Y. Ke, L. Ding, X. Wang, J. Yang, W. Zhang, K. P. Loh, F. Ding, Z. Liu, M. Chhowalla, Ultrathin transition metal oxychalcogenide catalysts for oxygen evolution in acidic media. *Nat. Synth.* **4**, 327–335 (2025).

10. A. Liu, H. Zhu, S. Bai, Y. Reo, M. Caironi, A. Petrozza, L. Dou, Y. Y. Noh, High-performance metal halide perovskite transistors. *Nat. Electron.* **6**, 559–571 (2023).

11. T. Y. Ko, H. Ye, G. Murali, S. Y. Lee, Y. H. Park, J. Lee, J. Lee, D. J. Yun, Y. Gogotsi, S. J. Kim, S. H. Kim, Y. J. Jeong, S. J. Park, I. In, Functionalized MXene ink enables environmentally stable printed electronics. *Nat. Commun.* **15**, 3459 (2024).

12. S. Pazos, K. Zhu, M. A. Villena, O. Alharbi, W. Zheng, Y. Shen, Y. Yuan, Y. Ping, M. Lanza, Synaptic and neural behaviours in a standard silicon transistor. *Nature* **640**, 69–76 (2025).

13. S. Wang, P. Zhou, Subnanosecond flash memory enabled by 2D-enhanced hot-carrier injection. *Nature* **641**, 90–97 (2025).

14. S. H. Choi, J. M. Sim, J. Shin, S. H. Ryu, T. Hwang, S. Y. Lim, H. J. Oh, J. H. Kwag, J. Y. Lee, K. C. Song, Y. Lee, M. Song, J. Kim, C. K. Park, Y. H. Song, J. S. Park, Unveiling the hybrid-channel (poly-Si/IGO) structure for 3D NAND flash memory for improving the cell current and GIDL-assisted erase operation. *Small Struct.* **6**, 2400495 (2025).

15. A. K. Geremew, S. Rumyantsev, M. A. Bloodgood, T. T. Salguero, A. A. Balandin, Unique features of the generation-recombination noise in quasi-one-dimensional van der Waals nanoribbons. *Nanoscale* **10**, 19749–19756 (2018).

16. J. Pavelka, J. Šikula, M. Tacano, M. Toita, Activation energy of RTS noise. *Radioengineering* **20**, 194–199 (2011).

17. Y. Wang, W. Duan, J. Meng, W. Zhang, Z. Liu, D. Guo, Z. Lv, J. Yu, H. Guan, A more random and secure image encryption method: A novel true random number generator based on a W/Ta2O5/Ag memristor. *J. Mater. Chem. C* **13**, 5920–5928 (2025).

18. Z. Ignjatovic, Y. Zhang, M. F. Bocko, CMOS image sensor readout employing in-pixel transistor current sensing. *Proc. IEEE Int. Symp. Circuits Syst.* , 1858–1861 (2008).

19. G. Datta, S. Kundu, Z. Yin, R. T. Lakkireddy, J. Mathai, A. P. Jacob, P. A. Beerel, A. R. Jaiswal, A processing-in-pixel-in-memory paradigm for resource-constrained TinyML applications. *Sci. Rep.* **12**, 14396 (2022).

20. W. Jin, P. C. H. Chan, S. Member, S. K. H. Fung, S. Member, P. K. Ko, Shot-noise-induced excess low-frequency noise in floating-body partially depleted SOI MOSFET's. *IEEE Trans. Electron. Devices* **46**, 1180–1185 (1999).

21. S. Ghosh, Z. E. Nataj, F. Kargar, A. A. Balandin, Electronic noise spectroscopy of quasi-two-dimensional antiferromagnetic semiconductors. *ACS Appl. Mater. Interfaces* **16**, 20920–20929 (2024).

22. C. G. Theodorou, E. G. Ioannidis, S. Haendler, E. Josse, C. A. Dimitriadis, G. Ghibaudo, Low frequency noise variability in ultra scaled FD-SOI n-MOSFETs: Dependence on gate bias, frequency and temperature. *Solid State Electron.* **117**, 88–93 (2016).

23. T. A. Kramer, R. F. W. Pease, Low frequency noise in sub-100 nm MOSFETs. *Phys. E Low Dimensional Syst. Nanostructures* **19**, 13–17 (2003).

24. F. Dieudonné, S. Haendler, J. Jomaah, F. Balestra, Low frequency noise in 0.12 μm partially and fully depleted SOI technology. *Microelectron. Reliab.* **43**, 243–248 (2003).

25. F. Dieudonné, S. Haendler, J. Jomaah, F. Balestra, Low frequency noise and hot-carrier reliability in advanced SOI MOSFETs. *Solid State Electron.* **48**, 985–997 (2004).

26. S. S. Choi, A. R. Choi, J. W. Yang, Y. W. Hwang, D. H. Cho, K. H. Shim, Comparative study of low frequency noise and hot-carrier reliability in SiGe PD SOI pMOSFETs. *Appl. Surf. Sci.* **254**, 6190–6193 (2008).

27. E. G. Ioannidis, S. Haendler, A. Bajolet, T. Pahron, N. Planes, F. Arnaud, R. A. Bianchi, M. Haond, D. Golanski, J. Rosa, C. Fenouillet-Beranger, P. Perreau, C. A. Dimitriadis, G. Ghibaudo, Low frequency noise variability in high-k/metal gate stack 28nm bulk and FD-SOI CMOS transistors. *Tech. Dig. Int. Electron Devices Meet. IEDM*, 18.6.1–18.6.4 (2011).

28. C. G. Theodorou, E. G. Ioannidis, F. Andrieu, T. Poiroux, O. Faynot, C. A. Dimitriadis, G. Ghibaudo, Low-frequency noise sources in advanced UTBB FD-SOI MOSFETs. *IEEE Trans. Electron Devices* **61**, 1161–1167 (2014).

29. C. G. Theodorou, E. G. Ioannidis, S. Haendler, N. Planes, F. Arnaud, J. Jomaah, C. A. Dimitriadis, G. Ghibaudo, Impact of front-back gate coupling on low frequency noise in 28 nm FDSOI MOSFETs. *Eur. Solid State Device Res. Conf.*, 334–337 (2012).

30. J. He, G. Li, Y. Lv, C. Wang, C. Liu, J. Li, D. Flandre, H. Chen, T. Guo, L. Liao, Defect self-compensation for high-mobility bilayer InGaZnO/In2O3 thin-film Transistor. *Adv. Electron. Mater.* **5**, 1900125 (2019).

31. C. G. Theodorou, A. Tsormpatzoglou, C. A. Dimitriadis, S. A. Khan, M. K. Hatalis, J. Jomaah, G. Ghibaudo, Origin of low-frequency noise in the low drain current range of bottom-gate amorphous IGZO thin-film transistors. *IEEE Electron Device Lett.* **32**, 898–900 (2011).

32. S. Jeon, S. Il Kim, S. Park, I. Song, J. Park, S. Kim, C. Kim, Low-frequency noise performance of a bilayer InZnOInGaZnO thin-film transistor for analog device applications. *IEEE Electron Device Lett.* **31**, 1128–1130 (2010).

33. C. Y. Jeong, J. I. Kim, J. H. Lee, J. G. Um, J. Jang, H. I. Kwon, Low-frequency noise properties in double-gate amorphous InGaZnO thin-film transistors fabricated by back-channel-etch method. *IEEE Electron Device Lett.* **36**, 1332–1335 (2015).

34. J. C. Park, S. W. Kim, C. J. Kim, S. Kim, D. H. Kim, I. T. Cho, H. I. Kwon, Low-frequency noise in amorphous indium-gallium-zinc oxide thin-film transistors from subthreshold to saturation. *Appl. Phys. Lett.* **97**, 2–5 (2010).

35. A. Tsormpatzoglou, N. A. Hastas, F. Mahmoudabadi, N. Choi, M. K. Hatalis, C. A. Dimitriadis, Characterization of high-current stress-induced instability in amorphous InGaZnO thin-film transistors by low-frequency noise measurements. *IEEE Electron Device Lett.* **34**, 1403–1405 (2013).

36. H. Lee, J. Yoo, H. Song, B. Lee, S. J. Yoon, S. Lim, J. H. Jeong, S. Kim, M. Park, S. Park, S. Jung, B. Pandit, T. Moon, J. H. Hwang, K. Lee, Y. K. Lee, K. Heo, H. Bae, Low-frequency noise and DC I-V characterization of gamma-ray irradiation-induced degradation and trap behaviors in a-IGZO TFTs. *Appl. Phys. Lett.* **126**, 062504 (2025).

37. N. Mavredakis, W. Wei, E. Pallecchi, D. Vignaud, H. Happy, R. Garcia Cortadella, A. Bonaccini Calia, J. A. Garrido, D. Jimenez, Velocity saturation effect on low frequency noise in short channel single layer graphene field effect transistors. *ACS Appl. Electron. Mater.* **1**, 2626–2636 (2019).

38. J. Renteria, R. Samnakay, S. L. Rumyantsev, C. Jiang, P. Goli, M. S. Shur, A. A. Balandin, Low-frequency 1/*f* noise in MoS_2 transistors: Relative contributions of the channel and contacts. *Appl. Phys. Lett.* **104**, 153104 (2014).

39. W. Shin, J. Byeon, R. H. Koo, J. Lim, J. H. Kang, A. R. Jang, J. H. Lee, J. J. Kim, S. N. Cha, S. Pak, S. T. Lee, Toward ideal low-frequency noise in monolayer CVD MoS_2 FETs: Influence of van der Waals junctions and sulfur vacancy management. *Adv. Sci.* **11**, 2307196 (2024).

40. X. Li, X. Xiong, T. Li, S. Li, Z. Zhang, Y. Wu, Effect of dielectric interface on the performance of MoS_2 transistors. *ACS Appl. Mater. Interfaces* **9**, 44602–44608 (2017).

41. J. W. Wang, Y. P. Liu, P. H. Chen, M. H. Chuang, A. Pezeshki, D. C. Ling, J. C. Chen, Y. F. Chen, Y. H. Lee, Controlled low-frequency electrical noise of monolayer MoS_2 with ohmic contact and tunable carrier concentration. *Adv. Electron. Mater.* **4**, 1700340 (2018).

42. N. Mavredakis, A. Pacheco-Sanchez, M. H. Alam, A. Guimerà-Brunet, J. Martinez, J. A. Garrido, D. Akinwande, D. Jiménez, Physics-based bias-dependent compact modeling of 1/*f* noise in single- to few-layer 2D-FETs. *Nanoscale* **15**, 6853–6863 (2023).

43. S. Nakaharai, T. Arakawa, A. Zulkefli, T. Iwasaki, K. Watanabe, T. Taniguchi, Y. Wakayama, Low-frequency noise in hBN/MoS_2/hBN transistor at cryogenic temperatures toward low-noise cryo-CMOS device applications. *Appl. Phys. Lett.* **122**, 262102 (2023).

44. D. C. Murray, A. G. R. Evans, J. C. Carter, Shallow defects responsible for GR noise in MOSFET's. *IEEE Trans. Electron Devices* **38**, 407–416 (1991).

45. B. Cretu, A. Bordin, E. Simoen, G. Hellings, D. Linten, C. Claeys, Detailed low frequency noise assessment on GAA NW n-channel FETs. *Solid State Electron.* **181-182**, 108029 (2021).

46. E. Simoen, B. Cretu, W. Fang, M. Aoulaiche, J. M. Routoure, R. Carin, S. dos Santos, J. Luo, C. Zhao, J. A. Martino, C. Claeys, Towards single-trap spectroscopy: Generation-recombination noise in UTBOX SOI nMOSFETs. *Phys. Status Solidi Curr. Top. Solid State Phys.* **12**, 292–298 (2015).

47. M. Jamal Deen, M. E. Levinshtein, S. L. Rumyantsev, J. Orchard-Webb, Generation-recombination noise in MOSFETs. *Semicond. Sci. Technol.* **14**, 298–304 (1999).

48. S. D. Dos Santos, B. Cretu, V. Strobel, J. M. Routoure, R. Carin, J. A. Martino, M. Aoulaiche, M. Jurczak, E. Simoen, C. Claeys, Low-frequency noise assessment in advanced UTBOX SOI nMOSFETs with different gate dielectrics. *Solid State Electron.* **97**, 14–22 (2014).

49. R. Talmat, H. Achour, B. Cretu, J. M. Routoure, A. Benfdila, R. Carin, N. Collaert, A. Mercha, E. Simoen, C. Claeys, Low frequency noise characterization in n-channel FinFETs. *Solid State Electron.* **70**, 20–26 (2012).

50. M. Andreev, J. Kang, T. Lee, J.-H. Park, "WSe_2 field-effect transistor with electron-beam-induced W-shaped I–V characteristic and its application to a ternary NAND gate," in *Proceedings of the 7th IEEE Electron Devices Technology and Manufacturing Conference (EDTM)* (IEEE, 2023); 10.1109/EDTM55494.2023.10103021.

51. M. Andreev, J. W. Choi, J. Koo, H. Kim, S. Jung, K. H. Kim, J. H. Park, Negative differential transconductance device with a stepped gate dielectric for multi-valued logic circuits. *Nanoscale Horizons* **5**, 1378–1385 (2020).

52. K. Thakar, S. Lodha, Multi-bit analog transmission enabled by electrostatically reconfigurable ambipolar and anti-ambipolar transport. *ACS Nano* **15**, 19692–19701 (2021).

53. B. Kim, Inkjet-printed indium oxide/carbon nanotube heterojunctions for gate-tunable diodes. *Adv. Electron. Mater.* **6**, 1901068 (2020).

54. S. Kim, S. Jung, B. Kim, H. Yoo, Pre-state-dependent ternary/binary logic operation obtained by inkjet printed indium oxide and single-walled carbon nanotube/indium oxide heterojunction-based transistors. *IEEE Electron Device Lett.* **44**, 265–268 (2023).

55. Z. Zhu, A. E. O. Persson, L. E. Wernersson, Reconfigurable signal modulation in a ferroelectric tunnel field-effect transistor. *Nat. Commun.* **14**, 2530 (2023).

56. R. Hayakawa, K. Takahashi, X. Zhong, K. Honma, D. Panigrahi, J. Aimi, K. Kanai, Y. Wakayama, Reconfigurable logic-in-memory constructed using an organic antiambipolar transistor. *Nano Lett.* **23**, 8339–8347 (2023).

57. C. Lee, J. Choi, H. Park, C. Lee, C. H. Kim, H. Yoo, S. G. Im, Systematic control of negative transconductance in organic heterojunction transistor for high-performance, low-power flexible ternary logic circuits. *Small* **17**, e2103365 (2021).

58. K. Kobashi, R. Hayakawa, T. Chikyow, Y. Wakayama, Interface engineering for controlling device properties of organic antiambipolar transistors. *ACS Appl. Mater. Interfaces* **10**, 2762–2767 (2018).

59. M. Huang, S. Li, Z. Zhang, X. Xiong, X. Li, Y. Wu, Multifunctional high-performance van der Waals heterostructures. *Nat. Nanotechnol.* **12**, 1148–1154 (2017).

60. X. Xiong, J. Kang, Q. Hu, C. Gu, T. Gao, X. Li, Y. Wu, Reconfigurable logic-in-memory and multilingual artificial synapses based on 2D heterostructures. *Adv. Funct. Mater.* **30**, 2–7 (2020).

61. L. Wu, W. Gao, Y. Sun, M. M. Yang, Z. Zheng, W. Fan, K. Shu, Z. Dan, N. Zhang, N. Huo, J. Li, Polarity-switchable and self-driven photo-response based on vertically stacked type-III GeSe/SnS_2 heterojunction. *Adv. Mater. Interfaces* **9**, 2102099 (2022).

62. G. B. Huang, M. Ramesh, T. Berg, E. Learned-Miller, Labeled faces in the wild: A database for studying face recognition in unconstrained environments. *Int. J. Comput. Vis.* **101**, 85–106 (2012).

63. L. Lopez, P. Masson, D. Née, R. Bouchakour, Temperature and drain voltage dependence of gate-induced drain leakage. *Microelectron. Eng.* **72**, 101–105 (2004).

64. S. Dutta, V. Kumar, A. Shukla, N. R. Mohapatra, U. Ganguly, Leaky integrate and fire neuron by charge-discharge dynamics in floating-body MOSFET. *Sci. Rep.* **7**, 8257 (2017).

65. A. A. Kadam, A. K. Singh, L. Somappa, M. S. Baghini, U. Ganguly, A compact low power multi-mode spiking neuron using band to band tunneling. *Proc. IEEE Int. Symp. Circuits Syst.* , 1–5 (2024).

66. T. Chavan, S. Dutta, N. R. Mohapatra, U. Ganguly, Band-to-band tunneling based ultra-energy-efficient silicon neuron. *IEEE Trans. Electron Devices* **67**, 2614–2620 (2020).

67. A. K. Singh, V. Saraswat, M. S. Baghini, U. Ganguly, Quantum tunneling based ultra-compact and energy efficient spiking neuron enables hardware SNN. *IEEE Trans. Circuits Syst. I Regul. Pap.* **69**, 3212–3224 (2022).

68. J. Kim, J. K. Han, H. Y. Maeng, J. Han, J. W. Jeon, Y. H. Jang, K. S. Woo, Y. K. Choi, C. S. Hwang, Fully CMOS-based p-bits with a bistable resistor for probabilistic computing. *Adv. Funct. Mater.* **34**, 2307935 (2024).

69. J. K. Han, J. Oh, G. J. Yun, D. Yoo, M. S. Kim, J. M. Yu, S. Y. Choi, Y. K. Choi, Cointegration of single-transistor neurons and synapses by nanoscale CMOS fabrication for highly scalable neuromorphic hardware. *Sci. Adv.* **7**, eabg8836 (2021).

70. S. Il Kim, H. J. You, M. S. Kim, U. S. An, M. S. Kim, D. H. Lee, S. T. Ryu, Y. K. Choi, Cryptographic transistor for true random number generator with low power consumption. *Sci. Adv.* **10**, eadk6042 (2024).

71. H. Mulaosmanovic, E. T. Breyer, T. Mikolajick, S. Slesazeck, Reconfigurable frequency multiplication with a ferroelectric transistor. *Nat. Electron.* **3**, 391–397 (2020).

72. Y. Cao, W. Liu, L. Qin, B. Liu, S. Chen, J. Ye, X. Xia, C. Wang, Entropy sources based on silicon chips: True random number generator and physical unclonable function. *Entropy* **24**, 1566 (2022).

73. H. D. Xiong, D. Heh, M. Gurfinkel, Q. Li, Y. Shapira, C. Richter, G. Bersuker, R. Choi, J. S. Suehle, Characterization of electrically active defects in high-k gate dielectrics by using low frequency noise and charge pumping measurements. *Microelectron. Eng.* **84**, 2230–2234 (2007).

74. E. Simoen, M. G. C. De Andrade, M. Aoulaiche, N. Collaert, C. Claeys, Low-frequency-noise investigation of n-channel bulk FinFETs developed for one-transistor memory cells. *IEEE Trans. Electron Devices* **59**, 1272–1278 (2012).

75. J. Jomaah, F. Balestra, G. Ghibaudo, Low frequency noise in advanced Si bulk and SOI MOSFETs. *J. Telecommun. Inf. Technol.* , 24–33 (2005).

76. T. Contaret, K. Romanjek, T. Boutchacha, G. Ghibaudo, F. Bœuf, Low frequency noise characterization and modelling in ultrathin oxide MOSFETs. *Solid State Electron.* **50**, 63–68 (2006).

77. I. Transactions, O. N. Electron, The kink-related excess low-frequency noise. *IEEE Trans. Electron Devices* **41**, 330–339 (1994).

78. T. Ushiki, H. Ishino, T. Ohmi, Effect of starting SOI material quality on low-frequency noise characteristics in partially depleted floating-body SOI MOSFETs. *IEEE Electron Device Lett.* **21**, 610–612 (2000).

79. H. M. Bu, Y. Shi, X. L. Yuan, Y. D. Zheng, S. H. Gu, H. Majima, H. Ishikuro, T. Hiramoto, Impact of the device scaling on the low-frequency noise in n-MOSFETs. *Appl. Phys. A Mater. Sci. Process.* **71**, 133–136 (2000).

80. S. Pathak, A. Amin, P. Srinivasan, F. Guarin, A. Dixit, "Impact of Chuck temperature on flicker noise (1/*f*) performance of PDSOI n-channel MOSFETs," in *2022 IEEE Latin American Electron Devices Conference (LAEDC)* (IEEE, 2022), pp. 1–4.

81. S. Pathak, S. Gupta, A. Rathi, P. Srinivasan, A. Dixit, Impact of gate oxide thickness on flicker noise (1/f) in PDSOI n-channel FETs. *Solid State Electron.* **217**, 108935 (2024).

82. C. G. Theodorou, E. G. Ioannidis, S. Haendler, N. Planes, E. Josse, C. A. Dimitriadis, G. Ghibaudo, "New LFN and RTN analysis methodology in 28 and 14nm FD-SOI MOSFETs," *2015 IEEE International Reliability Physics Symposium* (IEEE, 2015), pp. XT11–XT16.

83. O. Gauthier, S. Haendler, Q. Rafhay, C. Theodorou, Universality of trap-induced mobility fluctuations between 1/*f* noise and random telegraph noise in nanoscale FD-SOI MOSFETs. *Appl. Phys. Lett.* **122**, 233503 (2023).

84. L. Zafari, J. Jomaah, G. Ghibaudo, Low frequency noise in multi-gate SOI CMOS devices. *Solid State Electron.* **51**, 292–298 (2007).

85. C. A. Dimitriadis, F. V. Farmakis, G. Kamarinos, J. Brini, Origin of low-frequency noise in polycrystalline silicon thin-film transistors. *J. Appl. Phys.* **91**, 9919–9923 (2002).

86. Y. Liu, S. T. Cai, C. Y. Han, Y. Y. Chen, L. Wang, X. M. Xiong, R. Chen, Scaling down effect on low frequency noise in polycrystalline silicon thin-film transistors. *IEEE J. Electron Devices Soc.* **7**, 210–218 (2019).

87. C. T. Angelis, C. A. Dimitriadis, J. Brini, G. Kamarinos, V. K. Gueorguiev, T. E. Ivanov, Low-frequency noise spectroscopy of polycrystalline silicon thin-film transistors. *IEEE Trans. Electron Devices* **46**, 968–974 (1999).

88. Y. Yang, M. Zhang, L. Lu, M. Wong, H. S. Kwok, Low-frequency noise in bridged-grain polycrystalline silicon thin-film transistors. *IEEE Trans. Electron Devices* **69**, 1984–1988 (2022).

89. R. Koo, W. Shin, S. Kim, J. Kim, B. Kwak, J. Im, H. Kim, D. Kwon, S. S. Cheema, J. Lee, D. Kwon, Low-frequency noise spectroscopy for navigating geometrically varying strain effects in HfO_2 ferroelectric FETs. *Adv. Sci.* **12**, 2501367 (2025).

90. W. Shin, S. Kim, R. H. Koo, D. Kwon, J. J. Kim, D. H. Kwon, D. Kwon, J. H. Lee, Channel-length-dependent low-frequency noise characteristics of ferroelectric junctionless poly-Si thin-film transistors. *IEEE Electron Device Lett.* **44**, 1003–1006 (2023).

91. N. Mavredakis, R. G. Cortadella, X. Illa, N. Schaefer, A. B. Calia, J. A. Anton-Guimerà-Brunet, D. J. Garrido, D. Jiménez, Bias dependent variability of low-frequency noise in single-layer graphene FETs. *Nanoscale Adv.* **2**, 5450–5460 (2020).

92. J. Na, M. K. Joo, M. Shin, J. Huh, J. S. Kim, M. Piao, J. E. Jin, H. K. Jang, H. J. Choi, J. H. Shim, G. T. Kim, Low-frequency noise in multilayer MoS_2 field-effect transistors: The effect of high-k passivation. *Nanoscale* **6**, 433–441 (2014).

93. S. L. Rumyantsev, M. S. Shur, G. Liu, A. A. Balandin, “Low frequency noise in 2D materials: Graphene and MoS_2,” in *2017 International Conference on Noise and Fluctuations (ICNF)* (IEEE, 2017), pp. 1–4.

94. V. K. Sangwan, H. N. Arnold, D. Jariwala, T. J. Marks, L. J. Lauhon, M. C. Hersam, Low-frequency electronic noise in single-layer MoS_2 transistors. *Nano Lett.* **13**, 4351–4355 (2013).

95. A. Luque Rodríguez, J. A. Jiménez Tejada, S. Rodríguez-Bolívar, L. Mendes Almeida, M. Aoulaiche, C. Claeys, E. Simoen, Dependence of generation-recombination noise with gate voltage in FD SOI MOSFETs. *IEEE Trans. Electron Devices* **59**, 2780–2786 (2012).

96. D. S. Ang, Z. Lun, C. H. Ling, Generation-recombination noise in the near fully depleted SIMOX SOI n-MOSFET–Physical characteristics and modeling. *IEEE Trans. Electron Devices* **50**, 2490–2498 (2003).

97. I. Lartigau, J. M. Routoure, W. Guo, B. Cretu, R. Carin, A. Mercha, C. Claeys, E. Simoen, Low temperature noise spectroscopy of 0.1 μm partially depleted silicon on insulator metal-oxide-semiconductor field effect transistors. *J. Appl. Phys.* **101**, 104511 (2007).

98. F. N. Hooge, l/f Noise. *Proc. IEEE* **70**, 1926–1935 (1982).

99. R. F. Voss, J. Clarke, Flicker (1/f) noise: Equilibrium temperature and resistance fluctuations. *Phys. Rev. B* **13**, 556–573 (1976).

100. R. Adolph, *Low-Frequency Noise in Advanced Mos Devices* (Springer, 2016).

101. P. Gentil, S. Chausse, Low frequency noise measurements on silicon-on-sapphire (SOS) MOS transistors. *Solid State Electron.* **20**, 935–940 (1977).

102. W. Fang, E. Simoen, M. Aoulaiche, J. Luo, C. Zhao, C. Claeys, Distinction between silicon and oxide traps using single-trap spectroscopy. *Phys. Status Solidi Appl. Mater. Sci.* **212**, 512–517 (2015).

103. A. Asenov, “Random dopant induced threshold voltage fluctuations in quad-micron MOSFETs: A 3D ‘atomistic’ simulation study,” in *6th International Workshop on Computational Electronics (IWCE)* (IEEE, 1998), p. 263.

104. E. Lefeber, J. E. Rooda, in “*Handbook of Dynamic System Modeling*,” P. A. Fishwick, Ed. (Chapman & Hall/CRC, 2007), chap. 34.1.
